# Characterization of highly conserved G-quadruplex motifs as potential drug targets in *Streptococcus pneumoniae*

**DOI:** 10.1038/s41598-018-38400-x

**Published:** 2019-02-11

**Authors:** Subodh Kumar Mishra, Neha Jain, Uma Shankar, Arpita Tawani, Tarun Kumar Sharma, Amit Kumar

**Affiliations:** 1 Discipline of Biosciences and Biomedical Engineering, Indian Institute of Technology Indore, Simrol, Indore, 453552 India; 2 Centre for Bio-design and Diagnostics, Translational Health Science and Technology Institute, Faridabad, Haryana India

## Abstract

Several G-quadruplex forming motifs have been reported to be highly conserved in the regulatory regions of the genome of different organisms and influence various biological processes like DNA replication, recombination and gene expression. Here, we report the highly conserved and three potentially G-quadruplex forming motifs (SP-PGQs) in the essential genes (*hsdS*, *recD*, and *pmrA*) of the *Streptococcus pneumoniae* genome. These genes were previously observed to play a vital role in providing the virulence to the bacteria, by participating in the host-pathogen interaction, drug-efflux system and recombination- repair system. However, the presence and importance of highly conserved G-quadruplex motifs in these genes have not been previously recognized. We employed the CD spectroscopy, NMR spectroscopy, and electrophoretic mobility shift assay to confirm the adaptation of the G-quadruplex structure by the SP-PGQs. Further, ITC and CD melting analysis revealed the energetically favorable and thermodynamically stable interaction between a candidate G4 binding small molecule TMPyP4 and SP-PGQs. Next, TFP reporter based assay confirmed the regulatory role of SP-PGQs in the expression of PGQ harboring genes. All these experiments together characterized the SP-PGQs as a promising drug target site for combating the *Streptococcus pneumoniae* infection.

## Introduction

*Streptococcus pneumoniae* is one of the leading cause of death worldwide by causing pneumonia, meningitides, septicemia, and otitis media in ~1.6 million people per year, in both developed and developing countries^1,2^. In 2015, pneumococcal diseases caused 92,0136 deaths of children of five years or less, enumerating a total of 16% of all deaths of children worldwide. It is also the leading cause of community-acquired pneumonia(CAP) that has a high fatality rate and if survived by chance, causes ineradicable sequel development like neurological deficits, psychological impairment, and hearing loss^3^. Further, this notorious pathogen is also responsible for nosocomial (hospital-acquired) infections that are difficult to treat with the existing drug regimen^4^. Use of the conventional antibiotic drug is always under threat due to the drug resistance developing ability of the bacteria. *Streptococcus pneumoniae* has rapidly acquired resistance to optochin, sulphonamides, beta-lactams, lincosamides, erythromycin, trimethoprim-sulfamethoxazole, tetracycline, chloramphenicol macrolides, and fluoroquinolone^5^. The emergence of multi-drug resistant strains (MDR) and extremely resistant-strains (XDR) has worsened the pathological conditions. Therefore, to tackle these problems, there is an urgent and unmet need to identify a potential drug target in the evolutionary conserved drugable region of this pathogen that can act efficiently alike for both drug-susceptible and resistant strains.

**Figure 1:**
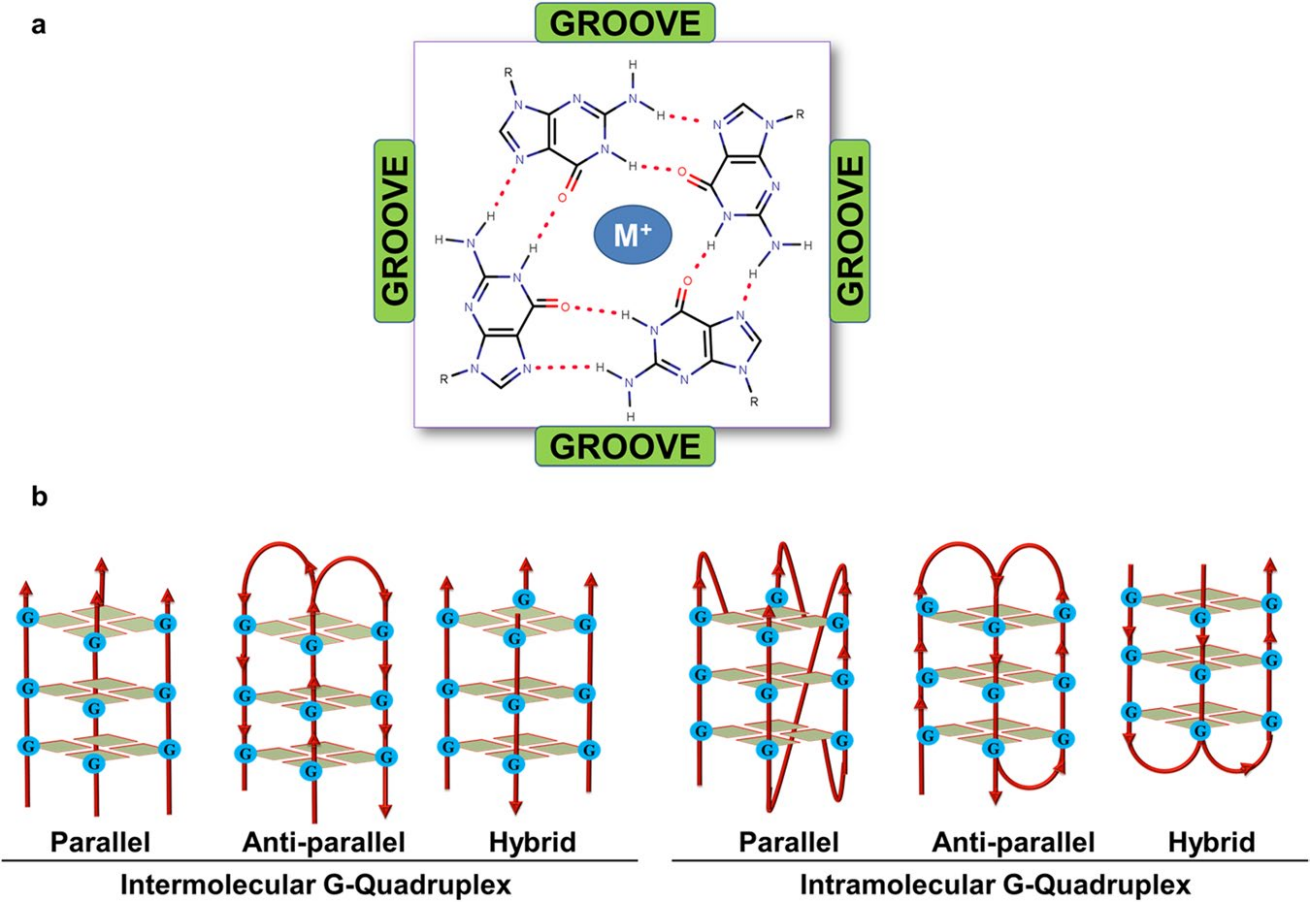
(**a**) G-quartet structure and (**b**) various topologies of G-quadruplex structures. M^+^ represents any cation or small molecule.

Guanine-rich nucleic acid sequences can fold into a distinct topology that is known as G-quadruplex (G4) structure^6,7^. The G-quadruplex structure can fold into diverse topologies depending upon the length of the G tract, length of the loop, the syn- and anti-conformation of Guanines. Figure 1 depicts the pattern of bond formation in a G-tetrad and the various kinds of topologies formed by the G-quadruplex structure. The G-quadruplexes are evolutionary conserved, highly stable secondary structures, and shown to orchestrate throughout the genome such as telomere, promoter of a various proto-oncogene, and regulatory regions of several essential genes of the humans and served as a potential target for cancer, developmental disorders, and neurological diseases^8^. Presence of the G-quadruplexes forming motif in the different locations of the genome functionally links with the regulation of replication, recombination, transcription, and translation process. For example, G-quadruplex regulates the transcription of a gene when present in the promoter region, and influence the replication when lies in the origin of replication. They have been observed for their active role in maintaining the genome stability when exists at the telomere^9^. G-quadruplex motif when present in the ORF (open reading frame) region of a gene, can halt the translation elongation *in vivo*^10,11^. There are several G-quadruplex interacting small molecule gone through the clinical trial for the treatment of various diseases^12^.

In the past few years, genome-wide mining of the G-quadruplex in the genome of human pathogens became a prime interest of investigators for developing an effective therapy against their infection. Recently, viruses are rapidly investigated for the presence of the G-quadruplex structure in their essential genes. For instances, the presence of the G-quadruplex forming motif in the long terminal repeat of the human immunodeficiency virus (HIV) genome was revealed to be crucial for the promoter function, and stabilization of this motif by a BRACO-19 inhibited the viral DNA replication^13,14^. The m-RNA of the EBNA1 protein of Epstein–Barr virus (EBV) showed to contain an inhibitory G-quadruplex forming motif and involved in escaping from the host immune system. Destabilization of this G-quadruplex motif restored the expression of the EBNA1 protein and increased the immuno-recognition of the virus by the host immune system^15^. The replication and transcriptional controlling genes (*oriLyt-R*, *oriLyt-L*, and *K5 9*, *v-IRF2* and *v-IRF3*) in the Kaposi’s sarcoma-associated herpesvirus (KSHV) genome showed to contain a G-quadruplex motif, and TMPyP4 and PhenDC3 were observed to reduce the virus latency by interacting with this G-quadruplex motifs^16^. Similarly, ICP8 (a critical factor in the virus replication) gene of Herpes Simplex Virus Type1 (HSV-1)^17^, preS2/S gene promoter of the Hepatitis B virus (HBV)^18^, the coding region of several genes (prM, E, NS1, NS3, NS5) of Zika virus genome^19^ contains G-quadruplex forming motif. These G-quadruplex motifs were shown to be involved in replication, transcription and translation regulation of the viral genome^14^. In a very recent study, Pac1 of human herpesvirus had been revealed to contain a G-quadruplex containing motif that is involved in concatemer cleavage during its replication and also required for packaging^20^. These studies have convincingly suggested the G-quadruplex as a promising drug target for the anti-viral therapy^20^.

Nevertheless, only a few classes of the bacteria have been studied so far for investigating the role of the G-quadruplex in their survival, propagation, and virulence. For example, the presence of the intramolecular G-quadruplex motif in the pilin expression locus (*pilE*) showed to provide the antigenic variation for the *Neisseria gonorrhoeae* bacteria^21^. *mce1R* operon that regulate the expression of the ATP-binding cassette transporter (ABC), genes coding for the protein of PE_PGRS family, Glucose-6-phosphate dehydrogenase 1 (*zwf1*), Oxidation-sensing Regulator Transcription Factor (*mosR*), membrane NADH dehydrogenase (*ndhA*), and ATP-dependent Clp protease (*clpx*) in *Mycobacterium tuberculosis* genome contains potential G-quadruplex motifs. All these genes showed to play an active role in providing virulence to the bacteria inside the host cell. Targeting these G-quadruplex motifs by G4 selective small molecule has been shown to reduce the survival and virulence of the bacteria^22,23^.

Considering, the ability of G-quadruplex as a promising drug target against human pathogenic infection, we sought to explore the potential G-quadruplex forming motifs (PGQs) in the *Streptococcus pneumoniae* genome. To the best of our knowledge, it is the first study that reports the presence of 3 different PGQs in the three essential genes: *hsdS*, *recD*, and *pmrA* in the *Streptococcus pneumoniae* genome that is found to be associated with the restriction-modification system, recombination, repair process, and drug efflux system, respectively^24–26^.

## Results

### Identification of G-quadruplex motifs in the *Streptococcus pneumoniae* genome

**Figure 2:**
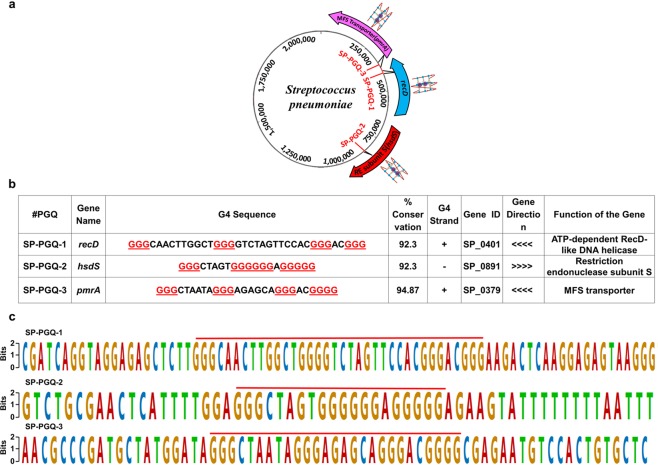
Essential PGQs in *Streptococcus pneumoniae* genome. (**a**) Schematic representation of essential PGQ sites in *Streptococcus pneumoniae* genome. (**b**) List of PGQ Sequences, percent of conservation, location, strand in which PGQ is present, and the gene in which the G-quadruplex is located. (≪≪- Antisense strand, ≫≫ Sense strand). (**c**) Weblogo representation of the highly conserved consensus sequence of the putative G-quadruplex sequences.

Considering the four consecutive runs of at least two guanine nucleotides with the gap of at least one loop forming nucleotide is required for the G-quadruplex formation *in vivo*^14^, here we searched for the potential G-quadruplex forming motifs (PGQs) in all available 39 completely sequenced strains of *Streptococcus pneumoniae* (Supplementary Table S1). We used our previously developed algorithm G4IPDB^27^ and confirmed the prediction once again by using the tools that were designed by other labs (Supplementary Info File S2, Supplementary Tables S2 and S3)^27–29^. G-quadruplex mining and Unweighted Pair Group Method with Arithmetic Mean (UPGMA) based clustering revealed three different highly conserved G-quadruplex structure motifs present in the open reading frame (ORF) of three essential genes (*hsdS*, *recD*, and *pmrA*) in the *Streptococcus pneumoniae* genome (Fig. 2 and Supplementary Table S4).

Though *S*. *pneumoniae* have only 39.7% GC content, its genome was found to be enriched in highly conserved PGQs in the essential genes, which undoubtedly suggests their essential role in the survival and pathogenicity of this bacteria. The conserved frequency of each PGQs was predicted using equation (1).

1 $$p\,({\rm{conserved}}\,{\rm{frequency}})=\frac{Number\,of\,strain\,in\,which\,{\rm{particular}}\,{\rm{PGQ}}\,{\rm{is}}\,{\rm{present}}}{Total\,number\,of\,strain(S.pneumonia)}\ast 100$$

Figure 2b describes the function, direction of the genes, strand of G-quadruplexes and conserved frequency of all predicted PGQs of *Streptococcus pneumonia* (SP-PGQs). Figure 2c describes the consensus sequence of SP-PGQs and 20 bp upstream/downstream from the PGQ motifs (Supplementary Tables S5–S7 showing conserved G-tract in the PGQ motifs).

SP-PGQ-1 found to present in the coding region of the *recD* gene that is an essential player of the RecBCD machinery and plays a vital role in the DNA double-strand break(DSBs) repair mechanism of the bacteria^30^. RecD protein has investigated for its role in the loading of RecA onto their target site (single-stranded DNA) and initiate the repair mechanism. Earlier, in *Neisseria gonnorheae*, it has been reported that G-quadruplex motif is essential for the recruitment of RecA and promotes strand exchange required for antigenic variations^31^. Therefore, inhibition of the expression of the *recD* gene will disturb the function of the RecA protein that eventually blocks the process of genetic exchange and DNA repair mechanism of the bacteria^30^. The stabilization of the PGQ motif present in the coding region of the RecD protein may inhibit its expression. Since, DNA double-stranded break is lethal to the bacteria, blocking the expression of key player of the bacterial DNA repair mechanism may evolve as a novel therapeutic strategy for combating the bacterial infection^30^.

Bioinformatics analysis revealed the presence of the SP-PGQ-2 in the coding region of the *hsdS* gene, that has observed for its essential role in the regulation of bacterial restriction-modification system type I (Type I RM)^24^. It encodes a specific protein that determines the target specificity of the other two component of the Type I RM system^24^. Type I RM system control the phase variation mechanism of the bacteria that involves the reversible transition of the bacterial colonization from opaque (more virulent in systemic infection) to transparent (less virulent) form. This phenotypic transition is crucial for the pneumococcal adaptation to different host niches and revealed to facilitate the pathogenesis^24^. Therefore, inhibition of the *hsdS* gene by stabilizing the PGQ motif will disrupt the pathogenesis mechanism of the bacteria and may represent another therapeutic approach for combating the *Streptococcus pneumonia* infection.

Bioinformatics analysis divulges the presence of the SP-PGQ-3 in the coding region of the *pmrA* gene. The *pmrA* gene encodes for a transmembrane protein that observed for their role in the drug efflux system of the bacteria and provides the drug resistance to the bacteria. It has been observed that a G-quadruplex motif present at the antisense strand regulate the gene expression at the transcription level^32^. However G-quadruplex present at the sense strand regulates the gene expression at translation level^32^. Interestingly, all the three SP-PGQs are present in the antisense strand of the gene, that works as the template strand during transcription. Thus, all the SP-PGQs would be able to regulate the expression of the PGQ harboring genes at the transcription level and represents a conserved drug target for developing active anti-bacterial drugs^32^.

### Circular Dichroism spectra and thermal denaturation analysis confirm the formation of stable G-quadruplex in the conserved PGQs

**Figure 3:**
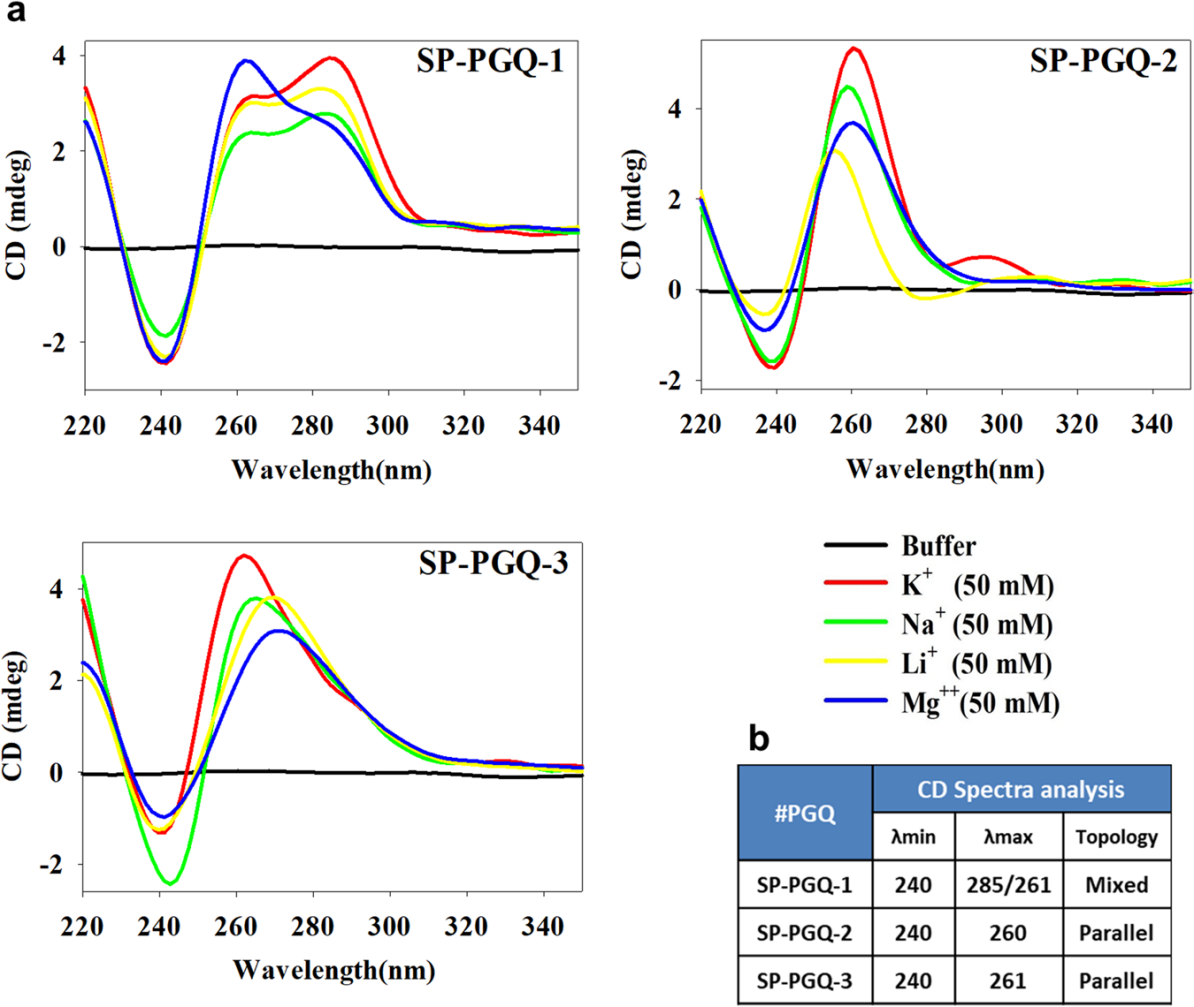
Circular Dichroism spectra analysis. (**a**) Spectrum of essential SP-PGQs in Tris-Cl buffer (10 mM) containing either of No cation (red), 50 mM KCl (green), 50 mM NaCl (yellow), 50 mM LiCl (blue) and 50 mM MgCl2 (magenta) (**b**) λ_max_/λ_min_ obtained from CD spectra analysis and the topologies of the respective PGQs in Tris-Cl buffer (10 mM) containing either of 50 mM KCl.

CD spectrophotometry was employed to verify the folding of the G-quadruplex structure by three PGQs (20 µM) in the presence of various ions (K^+^, Li^+^, Na^+^ and Mg^++^_,_ 50 mM each in Tris-Cl buffer, pH 7.2) (Fig. 3a). The previous studies have observed that positive peak at ~265 nm and a negative peak at ~240 nm represent parallel G-quadruplex while a positive peak at 290 nm and a negative peak at 260 nm represent an antiparallel G-quadruplex^33^. However, two positive peaks at 260 nm and 290 nm coupled with a negative peak at 240 nm represent a mixed or hybrid G-quadruplex structure^33^. Mixed or hybrid conformation may be due to the mixed conformation(Fig. 1) or due to the presence of both parallel and antiparallel G-quadruplex conformations in the sample. CD spectra analysis confirmed that SP-PGQ-1 forms hybrid G-quadruplex in all buffers. A similar pattern in the molecular ellipticity was observed with a smaller positive hump at 260 nm, and larger hump at 290 nm in the presence of K^+^, Na^+^ and Li^+^, but the reverse was observed in case of Mg^++^ ion as the larger hump shifted towards 260 nm and smaller at 290 nm. This can be due to the propensity of Mg^++^ to stabilize the parallel conformation in comparison to the anti-parallel one, thus giving larger peak at 260 nm (signature peak of parallel G4s) and a small peak at 290 nm (signature peak of anti-parallel G4s) and vice-versa for the other three cations. Interestingly, SP-PGQ-2 formed a hybrid structure in the presence of K^+^ ion by giving a minor peak at 290 nm. However, it folds into the parallel quadruplex topology of in the presence of other cations with the decreasing ellipticity in the order of Na^+^ > Mg^++^ > Li^+^ cation. SP-PGQ-3 showed parallel G-quadruplex conformation in the presence of K^+^, Na^+^ but shifted towards linear B-form in the presence of Li^+^ and Mg^++^ as the peak shifted towards ~270 nm. (Fig. 3b). Given that K^+^ ions selectively stabilizes the G-quadruplex folding by situating between the two consecutive G-tetrad and forming eight coordinate bonds with the carbonyl group of guanine residue, all SP-PGQs showed the highest stability in the buffer containing K^+^ ion.

To further check the effect of K^+^ ion concentration on the G-quadruplex stability, we performed the CD spectra in the absence of K^+^ cation and increasing concentration of K^+^ cation from 50 mM up to 200 mM (Supplementary Fig. S1). As anticipated, all the PGQs represented a direct proportionality in their stability and the CD molar ellipticity. Interestingly, SP-PGQ-03 were observed to exhibit the transition in the topology upon the increasing concentration of K^+^. Increasing K^+^ ion concentration converts it into the hybrid G-quadruplex structure from the parallel G-quadruplex structure (Supplementary Fig. S1).

**Figure 4:**
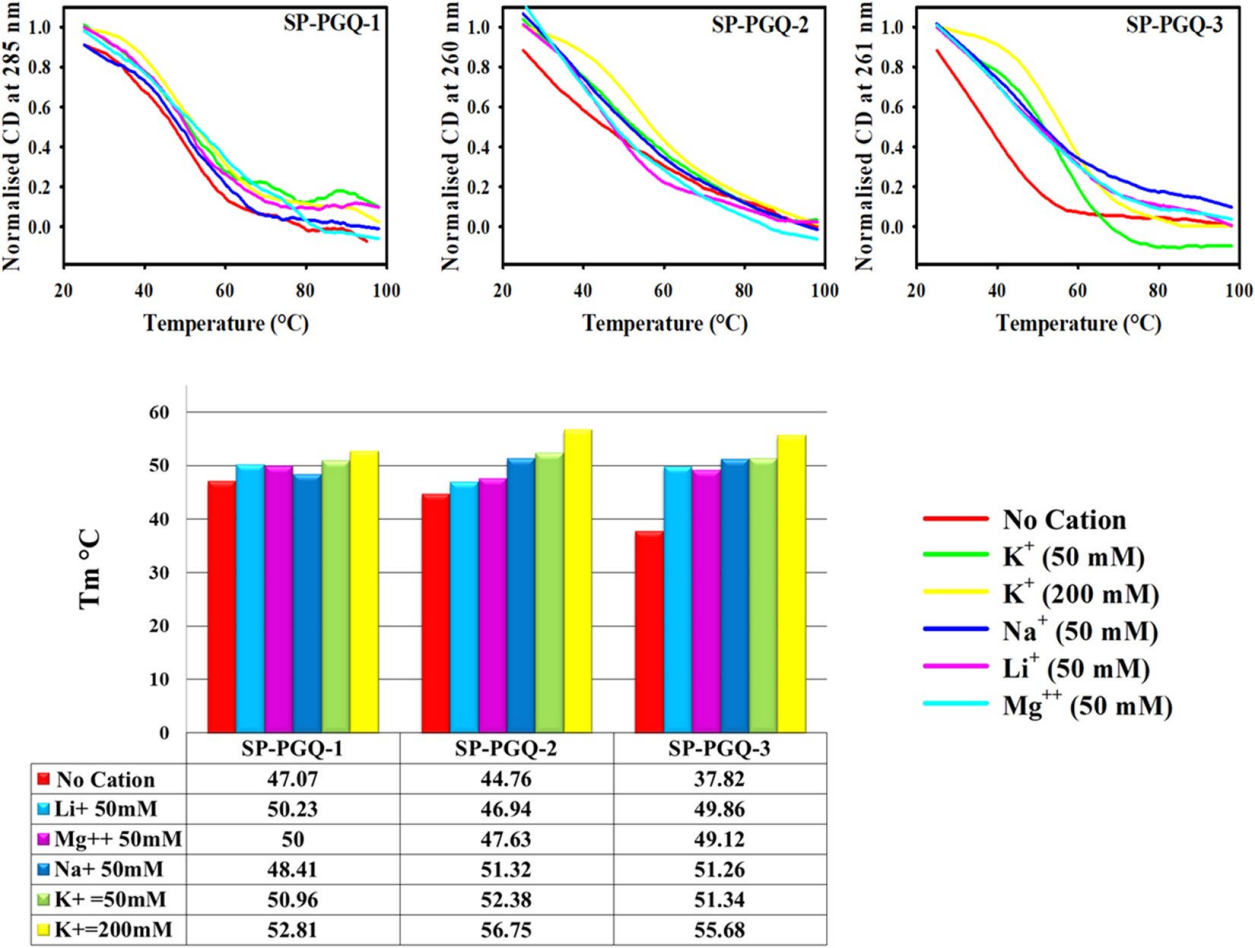
Thermal denaturation analysis. (**a**) CD Melting spectra of essential SP-PGQs in Tris-Cl buffer (10 mM) in the absence or presence of four different cations, No cation (red), 50 mM KCl (green), 200 mM KCl (yellow), 50 mM NaCl (blue), 50 mM LiCl (magenta) and 50 mM MgCl_2_ (fluorescent blue). (**b**) Bar graph depicting the melting temperatures in various buffers.

To assess the thermodynamic stability of PGQs, we performed CD melting analysis in the absence and presence of four cations [K^+^, Na^+^, Li^+^, Mg^++^] (Fig. 4a). Consistent with CD spectra studies, thermal denaturation analysis also showed the higher stability of G-quadruplex in the presence of K^+^ cation as compared with other cations (Fig. 4b and Supplementary Table S8).

### G-quadruplex formation confirmed by Nuclear Magnetic Resonance (NMR)

**Figure 5:**
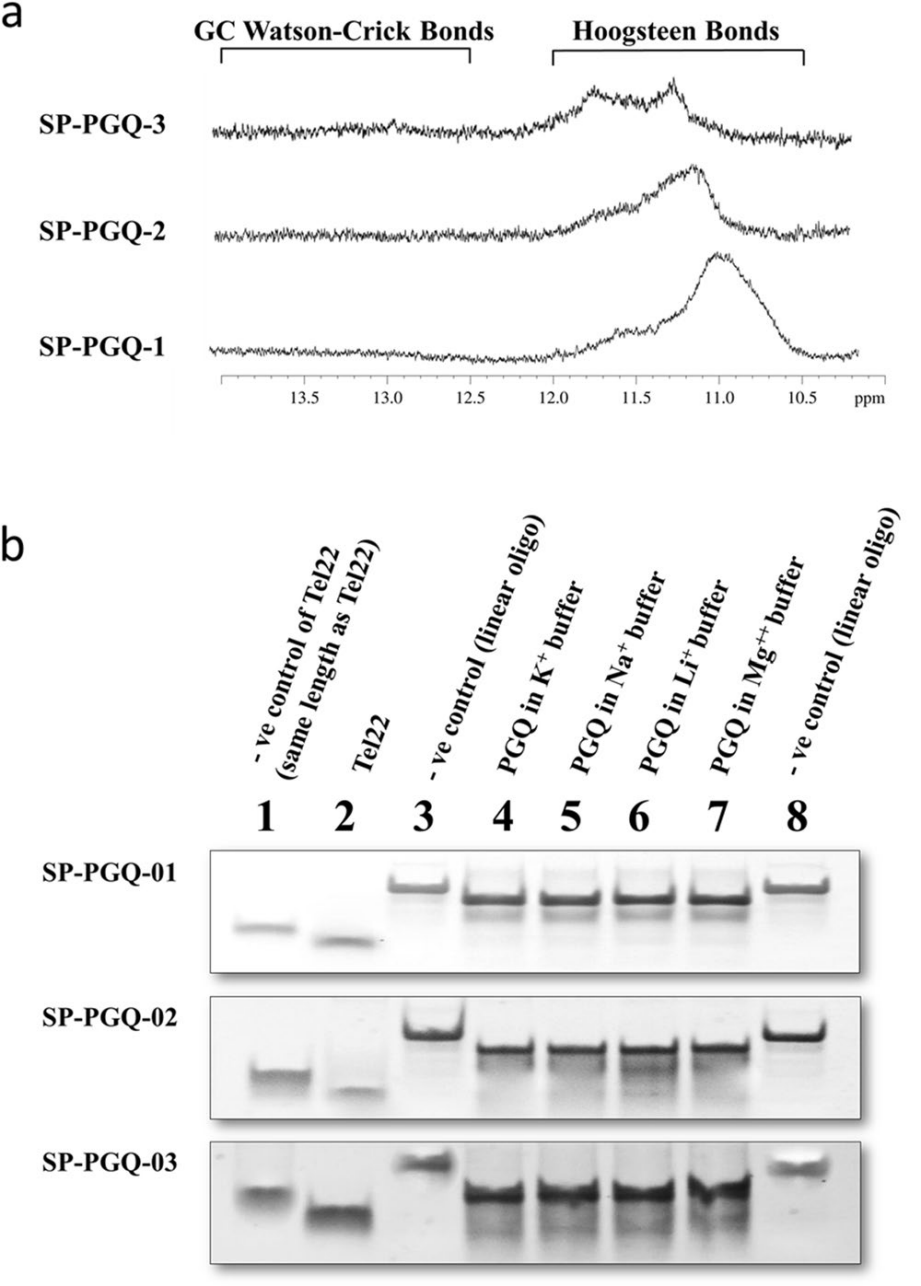
NMR Spectral and EMSA Analysis. (**a**) 1D ^1^H NMR spectra of the essential PGQs depicting imino protons involved in hoogsteen base pairing. (**b**) Electrophoretic shift shown by the SP-PGQs in comparison to their linear counterpart in Native PAGE. Cropped images are shown for each PGQs, and full-length gels are presented in Supplementary Fig. S2. All the three PGQs migrates faster as compared to there linear counterparts, depicting the formation of intramolecular G-quadruplexes.

We performed one-dimensional proton NMR (1D ^1^H NMR) experiments to validate the formation of G-quadruplex structure by SP-PGQs in the presence of K^+^ ion. In 1D ^1^H NMR, a chemical shift signal between 10–12 ppm represents a Hoogsteen hydrogen bonding between imino proton of guanine nucleotide of a G-quadruplex structure^34^. The chemical shift due to the Watson base paring appears between 12 to 14 ppm. All the SP-PGQs exhibited a chemical shift signal in between 12 to 14 ppm and evident the formation of the G-quadruplex structure by SP-PGQ-01, SP-PGQ-02, and SP-PGQ-03 (Fig. 5a).

### Gel retardation analysis strengthened the Intramolecular G-quadruplex formation

The electrophoretic mobility of the oligo sequences gives additional support to their molecularity and presence of multimeric conformation. The intramolecular forming G-quadruplex exhibits faster migration, while intermolecular forming G-quadruplex of the same length exhibits slower migration in the nondenaturing gel as compared to there linear counterparts^20^. Electrophoretic mobility shift assay of SP-PGQs were performed in the presence of four different cations in 1X TBE buffer along with a negative control (their linear counterparts) (Fig. 5b). See the Supplementary Fig. S3 for the gel image of an EMSA experiment performed separately in the presence of four different cation containing buffers. A well-known intramolecular G-quadruplex forming DNA, Tel22 was taken as a positive control. All the SP-PGQs migrated faster than their linear counterparts exhibiting the formation of stable intramolecular G-quadruplex conformations (Fig. 5b). Considering that intramolecular G-quadruplexes play essential roles as cis-regulatory site during the replication, recombination and gene expression^35^, intramolecular SP-PGQs may work as promising drug targets for developing a drug against *Streptococcus pneumoniae* Infection.

### Stabilizing and energetically favorable interaction of the PGQs with representative G4 ligand

Recently, G4 specific ligands have been studied for their therapeutic potential by affecting the stability of the G-quadruplex structure. TMPyP4 has been studied for its chemotherapeutic anticancer properties and observed to provide stability to human telomeric G-quadruplex^36^. TMPyP4 has successfully investigated for their inhibitory effect on expression of the L gene of the Ebola virus by stabilizing the G-quadruplex motif present in the L gene^37^. The L gene encodes for the viral RNA dependent RNA polymerase, and its inhibition stops the viral RNA processing inside the host cell^37^.

**Figure 6:**
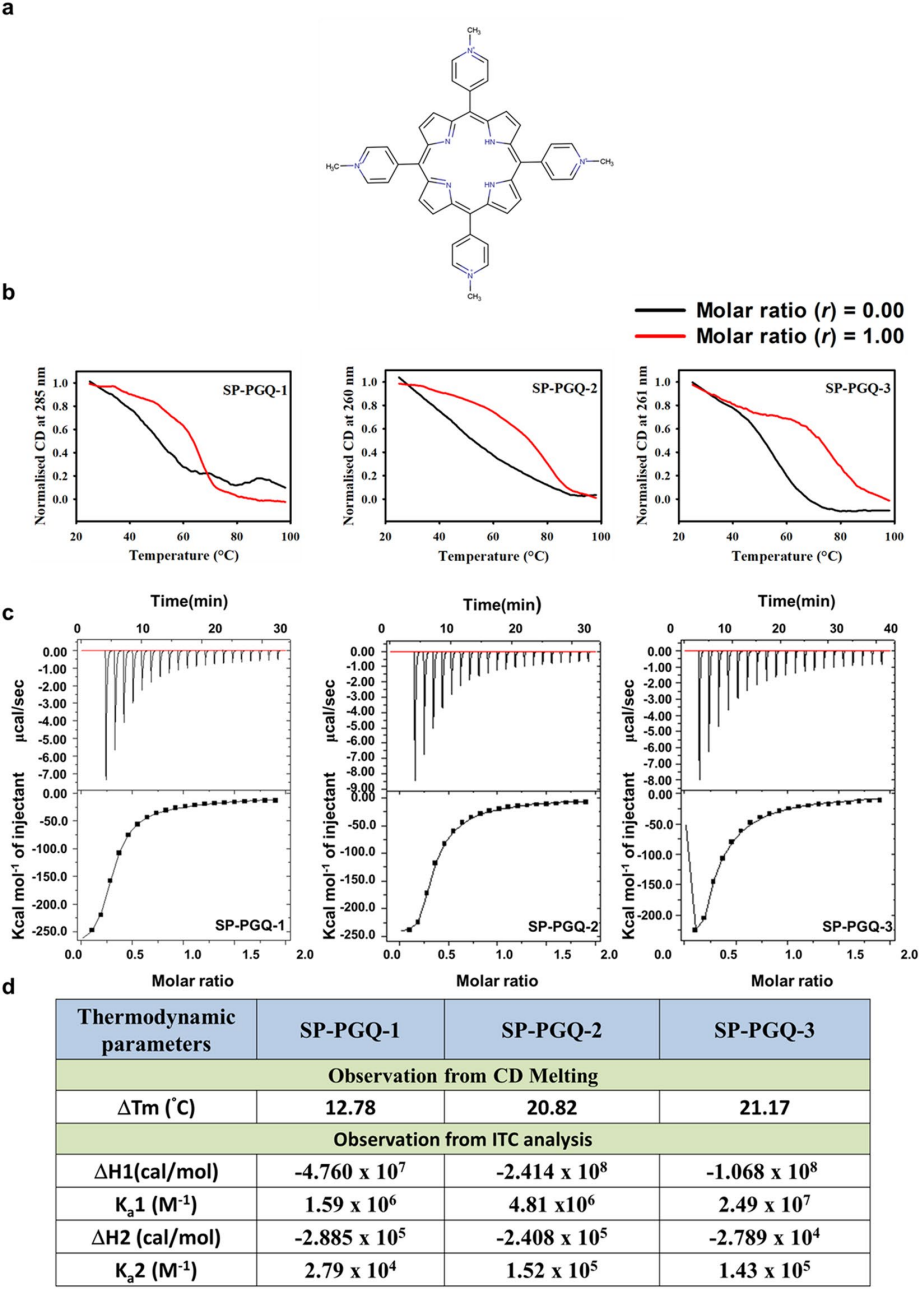
Interaction of SP-PGQs with TMPyP4. (**a**) 2D structure of TMPyP4 (**b**) Melting curves of SP-PGQs with TMPyP4 (**c**) ITC thermograms of SP-PGQs with TMPyP4. (**d**) List of thermodynamic parameters observed in CD melting and ITC analysis.

The above findings clearly indicate the potential of the TMPyP4 as a candidate therapeutics molecule to developed more clinically active antibacterial molecule. Therefore, here we extend our investigation to explore the stabilizing effect of TMPyP4 on the SP-PGQs (Fig. 6). As demonstrated in the Fig. 6b,d, binding of TMPyP4 to the PGQs result in the significant increase of the T_m_ of melting curve, which remarkably showed the stabilization of PGQs by the TMPyP4 at physiological ionic strength. To further evaluate the binding affinity of the TMPyP4 with PGQs, we performed the isothermal titration calorimetry (ITC)(Fig. 6c,d). The change in the enthalpy (ΔH_1_) for the high-affinity binding site of SP-PGQ-01, SP-PGQ-02 and SP-PGQ-03 were observed as −4.760 × 10^7^ cal/mol, −2.414 × 10^8^ cal/mol and −1.068 × 10^8^ cal/mol, respectively. The negative change in the enthalpy gives an observation of energetically favorable binding of the TMPyP4 with the SP-PGQs^38^. The association constant (K_a_) for the high binding site of SP-PGQ-1, SP-PGQ-2, and SP-PGQ-3 was observed as 1.59 × 10^6^ M^−1^, 4.81 × 10^6^ M^−1^ and 2.49 × 10^7^ M^−1^ respectively. We took TMPyP2 as control molecule for ITC experiments (see the Supplementary Fig. S4). ITC analysis gave a resilient observation that the binding of TMPyP4 with all PGQs was selective, energetically favorable phenomenon and TMPyP4 possessed the high affinity for all the PGQs (Fig. 6c,d).

### TMPyP4 stalls the Taq polymerase movement

To see the replication stalling ability of TMPyP4 by specific stabilization of G-quadruplex conformation, Taq polymerase stop assay was performed. The formation of stable G-quadruplex TMPyP4 complex inhibits the movement of Taq DNA polymerase, thereby decreasing the amplification rate. This leads to the demising band with the increasing concentration of TMPyP4 in the gel. The same was observed for all the three SP-PGQs (See the Supplementary Fig. S5) while TMPyP2, a structural isomer of TMPyP4, that have a low affinity of interacting with G-quadruplex conformation, showed a little or no effect on the taq polymerase arrest as indicated by the band intensities. A linear DNA that is unable to form G-quadruplex was used as a control that did not show any diminished band intensity effect while a slight decrease in the band intensities in the presence of TMPyP2 was observed depicting its non-specific binding with DNA. This effect shows the specific stabilization effect of G-quadruplex conformation by TMPyP4 and evident the formation of a stable complex with SP-PGQs.

### G-quadruplex stabilization decreases the mTFP expression

**Figure 7:**
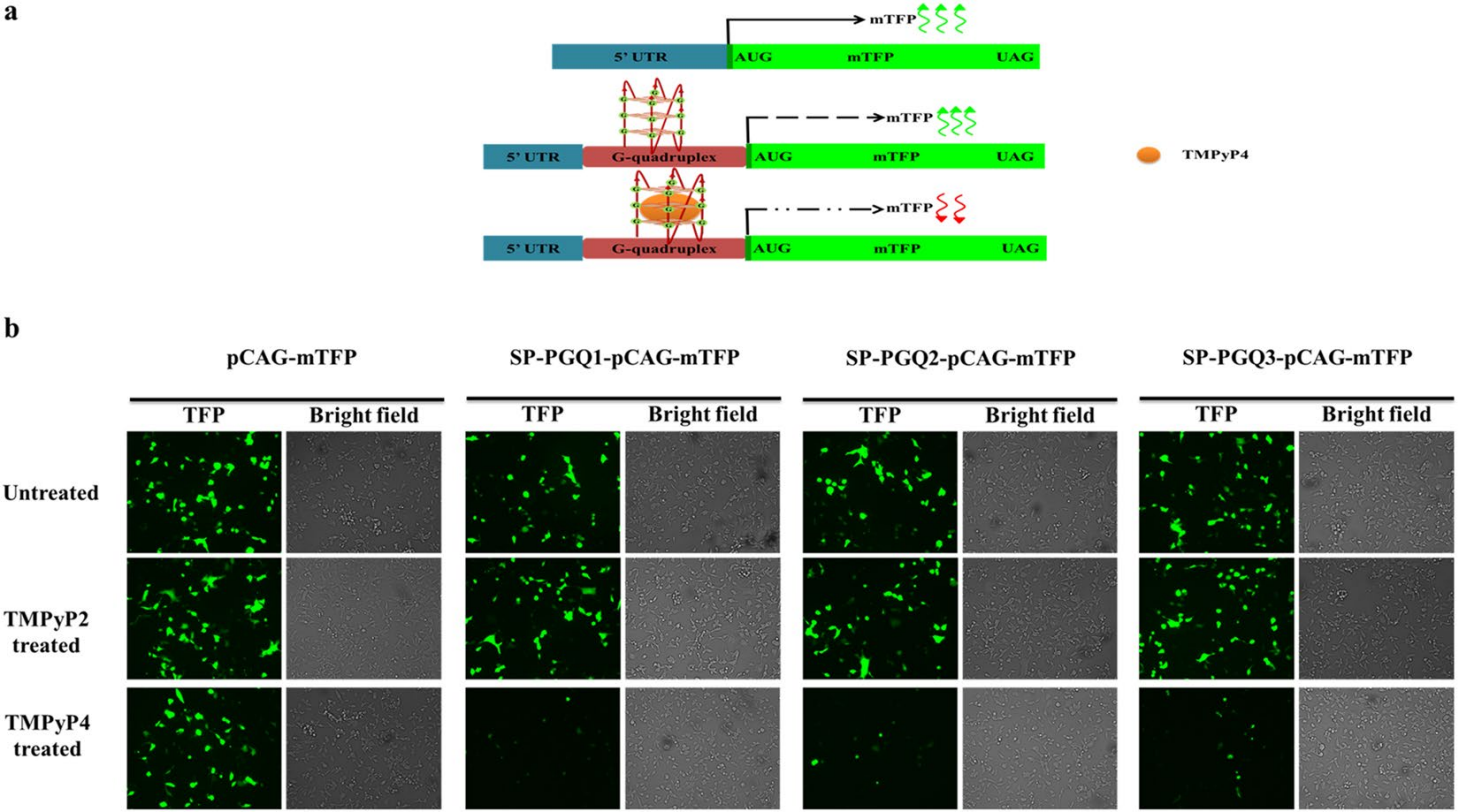
mTFP based Reporter assay. (**a**) Schematic representation of the native and the cloned plasmid and the effect on mTFP expression. the pCAG-mTFP plasmid was manually cloned to harbor the G4 motif at the 5′ UTR region of mTFP mRNA coding region. (**b**) TMPyP4 inhibits the mTFP expression by stabilizing the G-quadruplex motif cloned at the immediate upstream region of the ORF of mTFP protein in the pCAG plasmid, whereas, no inhibition was observed in the untreated or TMPyP2 treated HEK cell lines.

The stabilization of any secondary structure present in the gene region has been reported to inhibit the translation by inhibiting the movement of the transcriptional or translational machinery^10,39^. Therefore, to test this hypothesis, we fused the three G-quadruplex motifs (SP-PGQs) at the N terminus of the monomeric teal fluorescent reporter protein (mTFP) in the pCAG-mTFP plasmid by using overlap extension PCR cloning (Fig. 7). These modified plasmids were transfected in HEK 293 cell lines, and the expression of mTFP was analyzed under the fluorescence microscope. The mTFP expression is found to be inhibited significantly only in those cells that contain SP-PGQ- pCAG-mTFP plasmid and treated with TMPyP4 molecules. Whereas, TMPyP2 treatment did not cause any decrease in the expression of the mTFP reporter protein. The results of reporter-based assay is consistent with the previous results and suggested G4 mediated inhibition expression of the mTFP reporter protein.

## Discussion

Considering the G-quadruplex structure as encouraging drug target against pathogenic infection, we sought to explore the conserved G-quadruplex motifs in *Streptococcus pneumoniae* genome. Genome-wide bioinformatics analysis for all the 39 completely sequenced strain of *Streptococcus pneumoniae* revealed three potential G-quadruplex forming motifs (SP-PGQs) present in three different essential genes of bacteria.

Functional annotation of SP-PGQs suggested their crucial role in the survival and virulence of the bacteria. SP-PGQ-1 found to be located in the coding region of the *recD* gene that encodes for a heterodimeric ATP-dependent 5′-3′ DNA helicase. The *recD* homolog present in the *E*.*coli* was observed to be involved in the DNA recombination, double strand base repair, genome maintenance, and variation generation. Interestingly, previously it has been witnessed that Mutation in the RecD enzyme makes *Salmonella enterica* non-virulent and unable to propagate inside the macrophage^40^. Therefore, inhibition of *recD* expression may alter the virulence of *S*.*pneumoniae* bacteria as well^40,41^ although the additional studies are warranted in future that could provide experimental evidence in support of this potential hypothesis.

Very interestingly, Our bioinformatics analysis observed the presence of a conserved G-quadruplex forming motif (SP-PGQ-2) in the *hsdS* gene of the SpnD39III locus. SpnD39III locus comprised of the three con-transcribed genes (i) *hsdS* (ii) *hsdM* and (iii) *hsdR*^42^. The gene products of this locus make the component of Type I restriction-modification (Type I RM) that is well known as an immune system for the several classes of the bacteria. Recently, Type I RM has revealed to be involved in the phase variation mechanism of the *S*.*pneumoniae* that entail the reversible transition of the bacterial colony from opaque to transparent form^42^. This different colonization patterns are reversible in nature and holds different pathogenic characteristics. Opaque colonization contains a high amount of polysaccharide capsule and greater evasion of opsonophagocytic killing^24,43^. However, transparent colonies have the high amount of teichoic acid in its cell walls and process the high adherence properties of the epithelial cells^24^. The opaque variants of bacteria are deficient in the nasopharyngeal colonization but contain more virulence properties in the systemic infection. However, the transparent variants of bacteria display high nasopharyngeal colonization and low level of virulence^24^.

In the SpnD39III, *hsdR* codes for a DNA endonuclease, *hsdM* codes for a methyltransferase and *hsdS* code for a sequence specificity protein (S subunit) that govern the recognition site specificities for both endonuclease and methyltransferase^24,42^. Downstream to this locus a coding region for the *creX* DNA recombinase gene and two truncated hsdS genes (S1.SpnD39III and S2.SpnD39III) have been found^24,42^. This CreX DNA recombinase potentially generates the *hsdS* variants that produce the different target specificities for S subunit of Type I RM (Supplementary Fig. S6). This switching in the S subunit produce a different number, position, and type of methylation site in bacterial genome that caused to generate the opaque and transparent form of bacteria and plays a central role in providing adaptation to *S*.*pneumoniae* in different host niches^24,42^.

A highly conserved G-quadruplex motif (SP-PGQ-2) present in the *hsdS* gene of the SpnD39III locus suggested its essential role in the phenotypic transition of the bacteria from opaque (virulent) to transparent (less virulent forms) *S*.*pneumoniae*^24^. A G-quadruplex selective and stabilizing ligand would be able to bind to the G4 motif (SP-PGQ-2) present in the *hsdS* gene and inhibit their expression. Inhibited expression of the *hsdS* gene may inhibit this phase variation mechanism of bacteria and altered the virulence of the bacteria (Supplementary Fig. S6).

The Bioinformatics analysis revealed the presence of a conserved potential G-quadruplex motif (SP-PGQ-3) in the coding region of the MFS transporter gene, *pmrA* (SP_0379). The MFS transporter belongs to major facilitator superfamily (MFS) membrane protein. This protein is ubiquitously expressed in all life form and involved in the uptake of the nutrients and efflux of the antibiotics^26,44^. It comprised of 12 transmembrane spanning helixes and attributed to conferring drug resistance^45^. Their involvement in the transport of drugs and toxins, create the first line of defense for the bacteria against the anti-microbial agents^46^. Inhibition of expression of the MFS transporter by targeting the conserved G-quadruplex motif may represent another novel therapeutic approach to fight against *S*.*pneumoniae* infection^47^.

Next, to the functional annotation, G-quadruplex formations by SP-PGQs were confirmed by employing the 1D ^1^H-NMR. Circular dichroism spectroscopy (CD), and electrophoretic mobility shift assay (EMSA). 1D ^1^H-NMR confirmed the formation of the G-quadruplex structure by SP-PGQs; CD experiment confirmed the various topology of SP-PGQs in the presence of the different cations and EMSA confirmed the molecularity of the SP-PGQs.

Given the formation of the G-quadruplex structure by the SP-PGQs and the biological importance of the SP-PGQ harboring genes, it is essential to test the biological effects of stabilization of these SP-PGQs. Since TMPyP4 has already evaluated for their stabilizing effect on the G-quadruplex RNA present in the L gene of the Ebola virus, here we have evaluated the stabilizing of TMPyP4 on the SP-PGQs. CD melting analysis revealed the ∆T_m_ > 10 °C (difference in T_m_ between the presence and absence of TMPyP4) that signifies for the very well stabilizing effect of TMPyP4 on the SP-PGQs (Fig. 6b). To further test the affinity of TMPyP4 SP-PGQs we performed the ITC assay. In ITC assay we took a nonspecific G-quadruplex binding small molecule, TMPyP2 as a control. In comparison to the TMPyP2, TMPyP4 showed 2.79, 222 and 844 fold better affinity for the SP-PGQs (Fig. 6c and Supplementary Fig. S4). Further, to test the replication inhibition ability of TMPyP4 we performed the PCR stop assay in the absence and increasing concentration of the ligand. The analysis of PCR stop assay clearly evident the higher stabilization of the TMPyP4 in comparison to TMPyP2 on the movement of the polymerase enzyme and suggest the possible therapeutic approach to inhibit the growth of bacteria. We are further interested in seeing the change in the expression of PGQ harboring genes and therefore performed the reporter-based assay in the presence and absence of TMPyP4 and TMPyP2 molecule. The TFP based reporter assay confirmed the inhibiting effect of TMPyP4 on the expression of the PGQ possessing genes.

Several proteins have been observed to interact with cis-regulatory G-quadruplex structure and regulate the function by modulating their folding. For example, SgS1p of *Saccharomyces cerevisiae* and BLM helicase in human has shown to bind and unwind the topology of G-quadruplex structure^48,49^. RecQ helicase present in *Neisseria gonorrhoeae* unravel the G4 structures present in the pilin expression locus (*pilE*) and knocking down of RecQ helicase, inhibits the antigenic variation pathway in the pilin gene^50^. The G4 motif present upstream of the *pilE* gene of *Neisseria gonorreheae* acts as a DNA binding site for various recombination proteins including RecA^31^. Similarly, DinG, is a structure-specific multi-functional ATP helicase present in *Mycobacterium tuberculosis* resolves G4 secondary topology in the cell^23^. Therefore, we tried to find structural homologs of these helicases (RecA, DinG, and RecQ helicases) in *Streptococcus pneumoniae* genome. A blast analysis was performed for SgS1p, BLM, RecQ, RecA, and DinG against *Streptococcus pneumoniae* genome. Blast analysis revealed that the BLM and Sgs1p of human and Yeast are homologous to the DEAD/DEAH box family ATP-dependent RNA helicase protein of *Streptococcus pneumoniae* (Supplementary Figs S7 and S10a). RecQ and DinG of the *Streptococcus pneumoniae* shared significant domain similarity with the RecA of *Neisseria gonorrhoeae* and DinG of *Mycobacterium tuberculosis* respectively (Supplementary Figs S8–S10). This analysis suggested *Streptococcus pneumoniae* also encode for the similar kind of helicases that may interact with the cis-regulatory G4-quadruplex forming motif *in vivo* and regulate their activity.

In conclusion, the current study highlighted the three evolutionary conserved G-quadruplex motifs as a promising drug target that are present in the essential genes of bacteria providing virulence and drug resistance. Our observation of the presence of the conserved G4 motif in the S subunit of the Type I restriction-modification support the hypothesis of the regulatory role of the G4 structure in the virulence of the *Streptococcus pneumoniae*. A second interesting observation of the presence of the G4 motif in the coding region of MFS transporter and RecD provide additional evidence of the role of G4 structure in the *Streptococcus pneumonia* life cycle. Hence, pharmacological targeting of these conserved G4 motifs may suggest an alternative strategy for combating drug resistance problem in *Streptococcus pneumoniae* infection. A rational discovery of small molecule is anticipated that can selectively bind to these conserved G-quadruplex motifs and modulate their stability *in vivo*.

## Methods

### Genome sequence retrieval and prediction of G-quadruplex forming sequences in *Streptococcus pneumoniae*

Complete genome sequences of *Streptococcus pneumoniae* (Supplementary Table S1) were obtained from the NCBI Genome database (http://www.ncbi.nlm.nih.gov/genomes). G-quadruplex predictions were performed by using our previously published G-quadruplex prediction tool and reassessed by other available G-quadruplex mining tools, QGRS Mapper, and PQSFinder^27–29^. Our algorithm used the following standard expression [equation (2)] to explore the PGQ sequences in *S. pneumoniae*.

2 $${{\bf{G}}}_{{\boldsymbol{\{}}\mathrm{L1}{\boldsymbol{\}}}}[{\bf{N}}]{}_{{\boldsymbol{\{}}\mathrm{L2}{\boldsymbol{\}}}}{{\bf{G}}}_{{\boldsymbol{\{}}\mathrm{L1}{\boldsymbol{\}}}}\,[{\bf{N}}]{}_{{\boldsymbol{\{}}\mathrm{L2}{\boldsymbol{\}}}}{{\bf{G}}}_{{\boldsymbol{\{}}\mathrm{L1}{\boldsymbol{\}}}}\,[{\bf{N}}]{}_{{\boldsymbol{\{}}\mathrm{L2}{\boldsymbol{\}}}}{{\bf{G}}}_{{\boldsymbol{\{}}\mathrm{L1}{\boldsymbol{\}}}}$$

Where G refers to Guanine.

N = any nucleotide including Guanine.

L1 = length of consecutive Guanine tract set to more than or equal to 3.

L2 = Variable loop length ranges from 0 to 10.

This program explores both sense and antisense strands and looks for the putative G-quadruplex motif in the query sequence. Every prediction was listed and scrutinized for their location in NCBI GenBank. Further, sequences were aligned by using Clustal Omega. The alignments were then used for generating clustering tree by using the UPGMA algorithm. The consensus sequences for the most conserved PGQs with 20 bp upstream and downstream of the motifs were generated by using of DECIPHER tool.

### Genomic mapping and Functional annotation of the conserved PGQs

Genomic location of PGQs in the *Streptococcus pneumoniae* obtained from G-quadruplex prediction tool were mapped using the NCBI nucleotide database. Function and location of the G-quadruplexes were decisive by using Graphics mode of GeneBank database. Functions were annotated on the basis of the location: ORF or promoter region.

### Oligonucleotides and sample preparation

All the oligonucleotide sequences were purchased from Sigma Aldrich (Bangalore, India) and 100 μM stock solutions were prepared in MiliQ water according to the manufacturer protocol. Further dilution were made in Tris buffer (pH = 7.4, 10 mM) containing 50 mM of four different cations viz. K^+^, Na^+^, Li^+^ and Mg^++^ each. Before performing the experiment, samples were thermally denatured at 92 °C for 10 mins and cooled slowly to ambient temperature overnight. TMPyP4 was procured from Sigma Aldrich and used as such without further purification with the stock solution of 200 µM.

### Circular Dichroism and Melting experiment

CD spectra were recorded on Jasco J-815 Spectropolarimeter (Jasco Hachioji, Tokyo, Japan) with a Peltier junction temperature controller using a 1 mm path length quartz cuvette. A continuing supply of nitrogen gas was provided to prevent the water fortified around the cuvette. CD spectra for the PGQs were measured at 25 °C, over 220–320 nm and 20 nm/min scanning speed at the final concentration of 20 μM, in a 50 mM solution of four different cations (K^+^, Na^+^, Li^+^, and Mg^++^). Eventually, the background noise signal from the buffer spectrum were subtracted and zero corrected at 320 nm.

For melting temperature determination, spectra were obtained over a temperature range of 25 °C to 98 °C with the heating rate of 1 °C min-1 at the wavelength of the highest peak in the CD spectra of the individual PGQ. The change in absorbance was normalized at the respective wavelength vs. temperature and plotted using the SigmaPlot 12.0 software. Additionally, to check ligand binding interactions, melting analysis was performed with the increasing concentrations of TMPyP4. The CD spectra and melting analysis were performed in triplicate and the average values were considered for spectra and melting temperature analysis.

### Nuclear Magnetic Resonance

NMR experiments were performed by using AVANCE 400 MHz BioSpin International AG, Switzerland equipped with a 5 mm broadband inverse (BBI) probe. NMR data were processed and analyzed by using Topspin (1.3 version) software and 3 - (Trimethylsilyl) propionic-2, 2, 3, 3-D4 acid sodium salt (TSP) was taken as a reference compound. The analysis was performed in 90/10% H_2_O/D_2_O at 298 K with 20 ppm spectral width in potassium phosphate buffer. The experiments were performed in triplicates and the average values from the three experiments were used for analysis.

### Electrophoretic Mobility shift assay(EMSA)

A gel mobility shift assay was performed in 30% native polyacrylamide gel in 1X TBE buffer. The final concentration of 20 μM for each oligonucleotide in their respective cationic buffer (K^+^, Na^+^, Li^+^, and Mg^++^) was used. Initially, the 1X TBE buffer was used without any cation. Standard G-quadruplex DNA [Tel22, 5′-d(AGGGTTAGGGTTAGGGTTAGGG)-3′] was taken as a positive control and DNA oligonucleotides of the same length as that of PGQ as a negative control. Secondly, EMSA was performed for all the three SP-PGQs suspended in the four cations individually and run in the 1X buffer containing the respective cation. All gel assays were performed at 90 V in vertical gel unit at 4 °C and stained with EtBr for band visualization. ImageQuant LAS 4000 (GE Healthcare) was used for gel image analysis.

### Isothermal Titration Calorimetry (ITC)

ITC analysis was performed using a MicroCal iTC200 isothermal titration calorimeter (GE Healthcare) at 250C. PGQ oligonucleotides were dissolved in 10 mM potassium buffer (pH = 7.2). The stock solution of TMPyP4 was prepared in the same buffer condition. 21 injection of the 1.80 µL of TMPyP4 was added at each step from the syringe into the cell of the calorimeter containing PGQ oligonucleotides. Duration of each injection was set as 3.6 s and spacing between each successive injection was fixed at 90 s. Oligonucleotides heats of dilution were determined by injecting the same concentration of TMPyP4 into the potassium phosphate buffer and subtracted from the binding isotherms before curve fitting. Data were acquired in triplicate and analyzed by using origin scientific software version 7 (MicroCal Software Inc.) to generate thermograms. Thermograms were fitted in the two sites binding mode for association constant (Ka) determination.

### Taq polymerase PCR stop assay

Template for the three highly conserved PGQs (SP-PGQ1-3) templates, a linear control DNA, and the primers sharing the reverse complementarity with the last G-tracts (Supplementary Table S9) and Taq DNA polymerase were procured from Sigma-Aldrich Chemicals Ltd. St. Louis, MO, USA. The experiment was conducted in 25 µl reaction containing 1X PCR buffer, 4.25 mM MgCl_2_, 2 µM template, 0.33 mM dNTPs, 2.5 units of Taq polymerase followed by the dose titration (from 0–50 µM) of TMPyP4 or TMPyP2. PCR was performed in Prime Duo thermocycler (HiMedia) with the thermal cycle of an initial denaturation at 95 °C for 5 mins, 25 cycles of 95 °C for 30 s, 64 °C for 30 s and a final extension at 72 °C for 1 minute. The products of amplification were resolved on 3% agarose gel stained with ethidium bromide and visualized in ImageQuant LAS 4000 (GE Healthcare, Biosciences Ltd., Sweden).

### Construction of G4 harboring plasmid by overlap extension PCR (OE-PCR) cloning

G-quadruplex motifs (SP-PGQ1–3) were cloned by overlap extension PCR based cloning using overlapping forward and reverse primer in the N- terminus of mTFP(monomeric teal fluorescent protein) protein in the pCAG-mTFP plasmid [a gift from Dr. Debasis Nayak, IIT Indore] (Supplementary Table S10). PCR was performed by using Q5 polymerase enzyme in 25 µl reaction. The thermo-cyclic conditions were as follows: initial denaturation at 95 °C for 5 mins, annealing at 88.5 °C for 45 sec, and extension at 72 °C for 10 min. The extension and annealing were repeated for 30X cycles, and lastly, the samples were kept at 4 °C. The amplified products were treated with DpnI restriction enzyme (procured from NEB). DpnI specifically cleaves the host plasmid (methylated) but does not affect the non-methylated PCR products. These DpnI digested products were then transformed in DH5α strains of *E*. *coli*. A colony was picked from each plate and amplified in the DH5α culture media by growing overnight. Finally, the plasmids were isolated by using Midi-prep plasmid isolation kit (procured from Hi-media).

### HEK Cell Culture and plasmid transfection

Human embryonic kidney (HEK 293 procured from NCCS Pune, India) cells were maintained DMEM media containing 10% heat-denatured fetal bovine serum (FBS) as a supplement at 37 °C and 5% CO_2_ in a humidified incubator. Cells were grown and seeded in 6 well culture plates. At 60–75% cell confluency, cells were transfected with native pCAG-mTFP and the engineered plasmids by using Lipofectamine 3000 (procured from Invitrogen) as per the manufacturer’s protocol. The transfected cells were grown overnight and treated with TMPyP4 and TMPyP2 for 24 hours each. Expression of monomeric TFP (mTFP) was observed under fluorescence microscopy. The transfection and TFP expression analysis was performed in triplicate in order to avoid any false positive results or hand issues.

## Supplementary information

Supplemtary File S1

S2

## References

[1] Lynch JP, Zhanel GG (2009). Streptococcus pneumoniae: epidemiology, risk factors, and strategies for prevention. Semin. Respir. Crit. Care Med..

[2] Bandettini, R. & Melioli, G. Laboratory diagnosis of Streptococcus pneumoniae infections: past and future. *J*. *Prev*. *Med*. *Hyg*. **53** (2012). 23240165

[3] Koedel U, Scheld WM, Pfister H-W (2002). Pathogenesis and pathophysiology of pneumococcal meningitis. Lancet Infect. Dis..

[4] Jauneikaite E (2017). Nosocomial Outbreak of Drug-Resistant Streptococcus pneumoniae Serotype 9V in an Adult Respiratory Medicine Ward. J. Clin. Microbiol..

[5] Cherazard R (2017). Antimicrobial Resistant Streptococcus pneumoniae: Prevalence, Mechanisms, and Clinical Implications. Am. J. Ther..

[6] Kwok, C. K., Marsico, G. & Balasubramanian, S. Detecting RNA G-Quadruplexes (rG4s) in the Transcriptome. *Cold Spring Harb Perspect Biol* **10**, 10.1101/cshperspect.a032284 (2018). 10.1101/cshperspect.a032284 PMC6028067 29967010

[7] Burge S, Parkinson GN, Hazel P, Todd AK, Neidle S (2006). Quadruplex DNA: sequence, topology and structure. Nucleic Acids Res..

[8] Maizels N (2015). G4-associated human diseases. EMBO Rep..

[9] Bochman ML, Paeschke K, Zakian VA (2012). DNA secondary structures: stability and function of G-quadruplex structures. Nat. Rev. Genet..

[10] Endoh T, Kawasaki Y, Sugimoto N (2013). Suppression of gene expression by G-quadruplexes in open reading frames depends on G-quadruplex stability. Angew. Chem. Int. Ed. Engl..

[11] Murat P (2014). G-quadruplexes regulate Epstein-Barr virus–encoded nuclear antigen 1 mRNA translation. Nat. Chem. Biol..

[12] Balasubramanian S, Hurley LH, Neidle S (2011). Targeting G-quadruplexes in gene promoters: a novel anticancer strategy?. Nat. Rev. Drug Discov..

[13] Perrone R (2014). Anti-HIV-1 activity of the G-quadruplex ligand BRACO-19. J. Antimicrob. Chemother..

[14] Ruggiero E, Richter SN (2018). G-quadruplexes and G-quadruplex ligands: targets and tools in antiviral therapy. Nucleic Acids Res..

[15] Lista MJ (2017). Nucleolin directly mediates Epstein-Barr virus immune evasion through binding to G-quadruplexes of EBNA1 mRNA. Nat. Commun..

[16] Madireddy A (2016). G-quadruplex-interacting compounds alter latent DNA replication and episomal persistence of KSHV. Nucleic Acids Res..

[17] Artusi S (2015). The Herpes Simplex Virus-1 genome contains multiple clusters of repeated G-quadruplex: Implications for the antiviral activity of a G-quadruplex ligand. Antiviral Res..

[18] Biswas B, Kandpal M, Vivekanandan P (2017). A G-quadruplex motif in an envelope gene promoter regulates transcription and virion secretion in HBV genotype B. Nucleic Acids Res..

[19] Fleming AM, Ding Y, Alenko A, Burrows CJ (2016). Zika virus genomic RNA possesses conserved G-quadruplexes characteristic of the flaviviridae family. ACS Inf. Dis..

[20] Biswas, B., Kumari, P. & Vivekanandan, P. Pac1 Signals of Human Herpesviruses Contain a Highly Conserved G-Quadruplex Motif. *ACS Inf*. *Dis* (2018). 10.1021/acsinfecdis.7b00279 29493219

[21] Cahoon LA, Seifert HS (2009). An alternative DNA structure is necessary for pilin antigenic variation in Neisseria gonorrhoeae. Science.

[22] Perrone R (2017). Mapping and characterization of G-quadruplexes in Mycobacterium tuberculosis gene promoter regions. Sci. Rep..

[23] Thakur RS (2014). Mycobacterium tuberculosis DinG Is a Structure-specific Helicase That Unwinds G4 DNA Implications ofr Targeting G4 DNA as a Novel Therapeutic Approach. J. Biol. Chem..

[24] Li J (2016). Epigenetic Switch Driven by DNA Inversions Dictates Phase Variation in Streptococcus pneumoniae. PLoS Path..

[25] Smith GR (2012). How RecBCD enzyme and Chi promote DNA break repair and recombination: a molecular biologist’s view. Microbiol. Mol. Biol. Rev..

[26] Gill MJ, Brenwald NP, Wise R (1999). Identification of an efflux pump gene, pmrA, associated with fluoroquinolone resistance in Streptococcus pneumoniae. Antimicrob. Agents Chemother..

[27] Mishra SK, Tawani A, Mishra A, Kumar A (2016). G4IPDB: A database for G-quadruplex structure forming nucleic acid interacting proteins. Sci. Rep..

[28] Kikin O, D’Antonio L, Bagga PS (2006). QGRS Mapper: a web-based server for predicting G-quadruplexes in nucleotide sequences. Nucleic Acids Res..

[29] Hon J, Martinek T, Zendulka J, Lexa M (2017). pqsfinder: an exhaustive and imperfection-tolerant search tool for potential quadruplex-forming sequences in R. Bioinformatics.

[30] Dillingham MS, Kowalczykowski SC (2008). RecBCD Enzyme and the Repair of Double-Stranded DNA Breaks. Microbiol. Mol. Biol. Rev..

[31] Kuryavyi V, Cahoon LA, Seifert HS, Patel DJ (2012). RecA-binding pilE G4 sequence essential for pilin antigenic variation forms monomeric and 5′ end-stacked dimeric parallel G-quadruplexes. Structure.

[32] Agarwal T, Roy S, Kumar S, Chakraborty TK, Maiti S (2014). In the sense of transcription regulation by G-quadruplexes: asymmetric effects in sense and antisense strands. Biochemistry..

[33] Kypr J, Kejnovská I, Renčiuk D, Vorlíčková M (2009). Circular dichroism and conformational polymorphism of DNA. Nucleic Acids Res..

[34] Adrian M, Heddi B, Phan AT (2012). NMR spectroscopy of G-quadruplexes. Methods.

[35] Parrotta L (2014). Targeting unimolecular G-quadruplex nucleic acids: a new paradigm for the drug discovery?. Expert Opin. Drug Discov..

[36] Zheng XH (2016). TMPyP4 promotes cancer cell migration at low doses, but induces cell death at high doses. Sci. Rep..

[37] Wang SR (2016). Chemical Targeting of a G-Quadruplex RNA in the Ebola Virus L Gene. Cell Chem. Biol..

[38] Du X (2016). Insights into Protein–Ligand Interactions: Mechanisms, Models, and Methods. Int. J. Mol. Sci..

[39] Holder IT, Hartig JS (2014). A matter of location: influence of G-quadruplexes on Escherichia coli gene expression. Chem. Biol..

[40] Cano DA, Pucciarelli MG, García-del Portillo F, Casadesús J (2002). Role of the RecBCD Recombination Pathway in Salmonella Virulence. J. Bacteriol..

[41] Regha K, Satapathy AK, Ray MK (2005). RecD Plays an Essential Function During Growth at Low Temperature in the Antarctic Bacterium Pseudomonas syringae Lz4W. Genetics..

[42] Manso AS (2014). A random six-phase switch regulates pneumococcal virulence via global epigenetic changes. Nat. Commun..

[43] Weiser JN, Markiewicz Z, Tuomanen EI, Wani JH (1996). Relationship between phase variation in colony morphology, intrastrain variation in cell wall physiology, and nasopharyngeal colonization by Streptococcus pneumoniae. Infect. Immun..

[44] Hasdemir U (2007). [The role of cell wall organization and active efflux pump systems in multidrug resistance of bacteria]. Mikrobiyol. Bul..

[45] Pao SS, Paulsen IT, Saier MH (1998). Major facilitator superfamily. Microbiol. Mol. Biol. Rev..

[46] Rahman T, Yarnall B, Doyle DA (2017). Efflux drug transporters at the forefront of antimicrobial resistance. Eur. Biophys. J..

[47] Kumar S (2016). Bacterial Multidrug Efflux Pumps of the Major Facilitator Superfamily as Targets for Modulation. Infect. Disord. Drug Targets..

[48] Sun H, Karow JK, Hickson ID, Maizels N (1998). The Bloom’s syndrome helicase unwinds G4 DNA. J. Biol. Chem..

[49] Sun H, Bennett RJ, Maizels N (1999). The Saccharomyces cerevisiae Sgs1 helicase efficiently unwinds G-G paired DNAs. Nucleic Acids Res..

[50] Cahoon LA, Manthei KA, Rotman E, Keck JL, Seifert HS (2013). Neisseria gonorrhoeae RecQ helicase HRDC domains are essential for efficient binding and unwinding of the pilE guanine quartet structure required for pilin antigenic variation. J. Bacteriol..

