# Supplementary material for: Characterization of highly conserved G-quadruplex motifs as potential drug targets in Streptococcus pneumoniae
Source: Sci Rep. 2019 Feb 11;9:1791. doi: 10.1038/s41598-018-38400-x (PMC6370756; doi:10.1038/s41598-018-38400-x)
Supplement: Supplementary file 1 — Supplemtary File S1 [file 41598_2018_38400_MOESM1_ESM.pdf]

Characterization of highly conserved G-quadruplex motifs as potential drug targets in *Streptococcus pneumoniae*

Subodh Kumar Mishra[1], Neha Jain[1], Uma Shankar[1], Arpita Tawani[1], Tarun Kumar Sharma[2], Amit Kumar[1*]

[1]Discipline of Biosciences and Biomedical Engineering, Indian Institute of Technology Indore, Simrol, Indore 453552, India.

[2]Centre for Bio-design and Diagnostics, Translational Health Science and Technology Institute, Faridabad, Haryana, India.

*Corresponding author - Dr. Amit Kumar. Tel: +91-731-2438771; Fax: +91-731-2438721; Email: amitk@iiti.ac.in

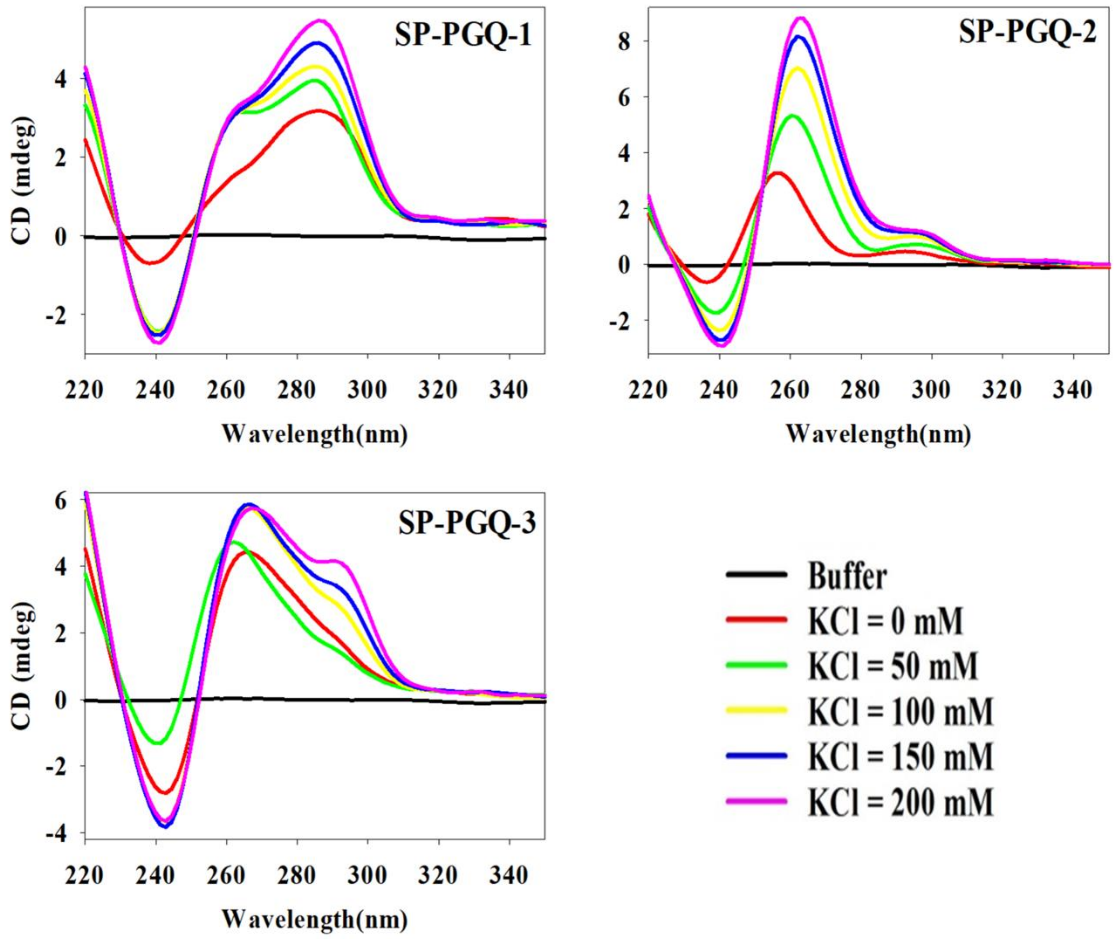

Supplementary Figure S1. CD of SP-PGQs in K+ Buffer. Circular Dichroism spectra of conserved SP-PGQs in Tris-Cl buffer (10 mM) containing an increasing concentration of K+ cation, 0 mM KCl (red), 50 mM KCl (green), 100 mM KCl (yellow), 150 mM KCl (blue) and 200 mM KCl (magenta)

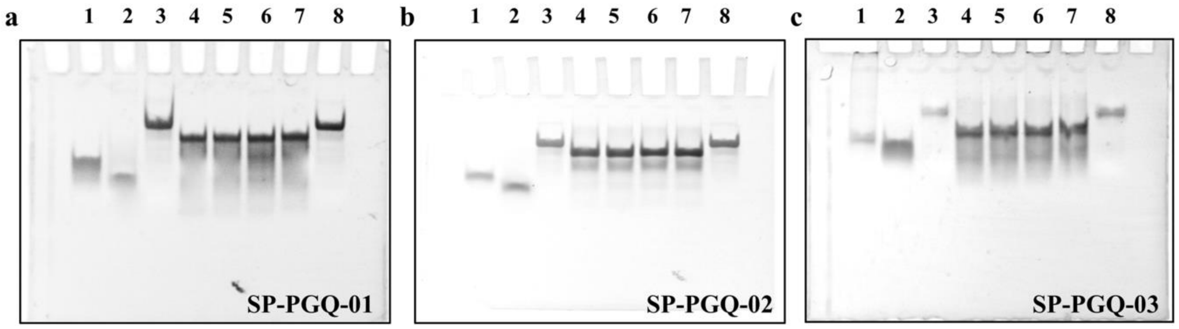

Supplementary Figure S2: Full length gel images of Figure 5b. Lane 1: - ve control of Tel22 (same length as Tel22); Lane 2: Positive control (Tel22); Lane 3: Negative control of PGQ (linear DNA of comparable length with PGQs); Lane 4: PGQ in K^+ buffer; Lane 5: PGQ in Na^+ buffer; Lane 6: PGQ in Li^+ buffer; Lane 7: PGQ in Mg^{2+} buffer; 8: Negative control of PGQ (linear DNA of comparable length with PGQs).

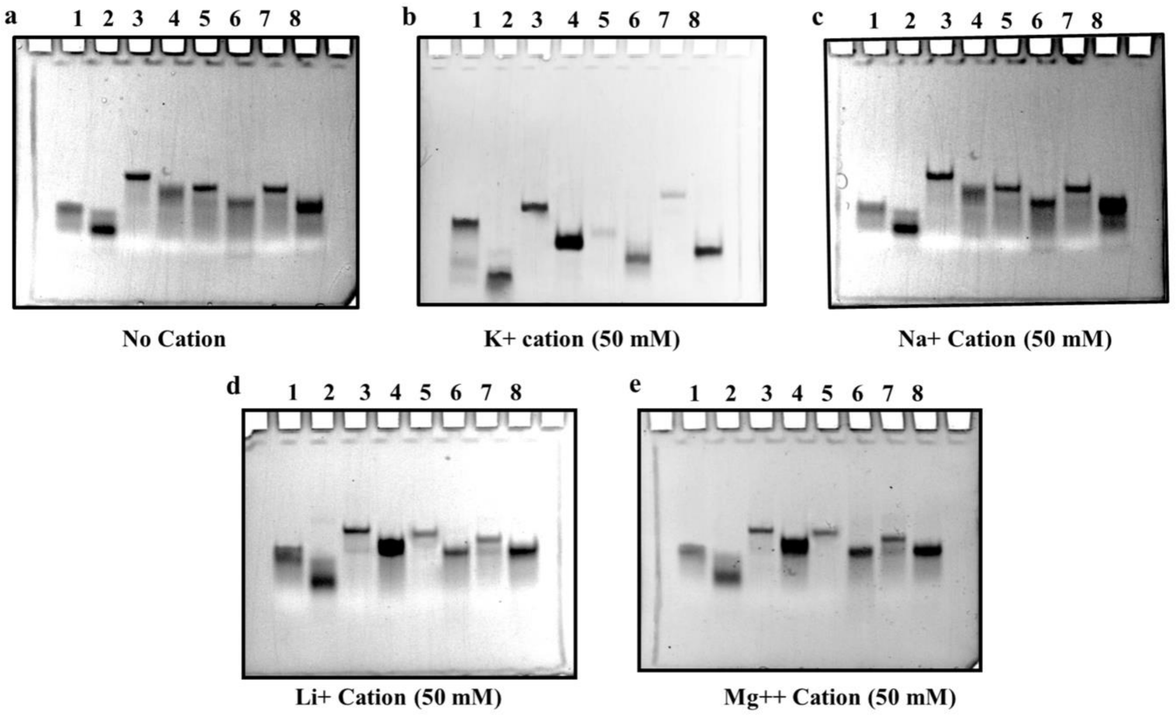

Supplementary Figure S3: EMSA of SP-PGQs in the absence and presence of four cations and cationic buffers. Electrophoretic mobility shift assay for SP-PGQs dissolved in a specific cation and run on the buffer containing the same cation. Lane 1: : - ve control of Tel22 (same length as Tel22); Lane 2: Positive control (Tel22); Lane 3: Negative control of SP-PGQ-1(linear DNA of comparable length with SP-PGQ-1); Lane 4: SP-PGQ-1 in the absence or the presence of cation buffer; Lane 5: Negative control of SP-PGQ-2(linear DNA of comparable length with SP-PGQ-1); Lane 6: SP-PGQ-2 in the absence or the presence of cationic buffer; Lane 7: Negative control of SP-PGQ-3(linear DNA of comparable length with SP-PGQ-3); Lane 8: SP-PGQ-3 in the absence or the presence of cation buffer. a) in the absence of any cation run on the only 1X TBE buffer. b) SP-PGQs dissolved in 50 mM K+ ion run on gel in 1X TBE buffer containing 50 mM K^+. c) SP-PGQs dissolved in 50 mM Na+ ion run on gel in 1X TBE buffer containing 50 mM Na^+. d) SP-PGQs dissolved in 50 mM Li^+ ion run on gel in 1X TBE buffer containing 50 mM Li^{+}. e) SP-PGQs dissolved in 50 mM Mg^{++} ion run on gel in 1X TBE buffer containing 50 mM Mg^{++}.

a

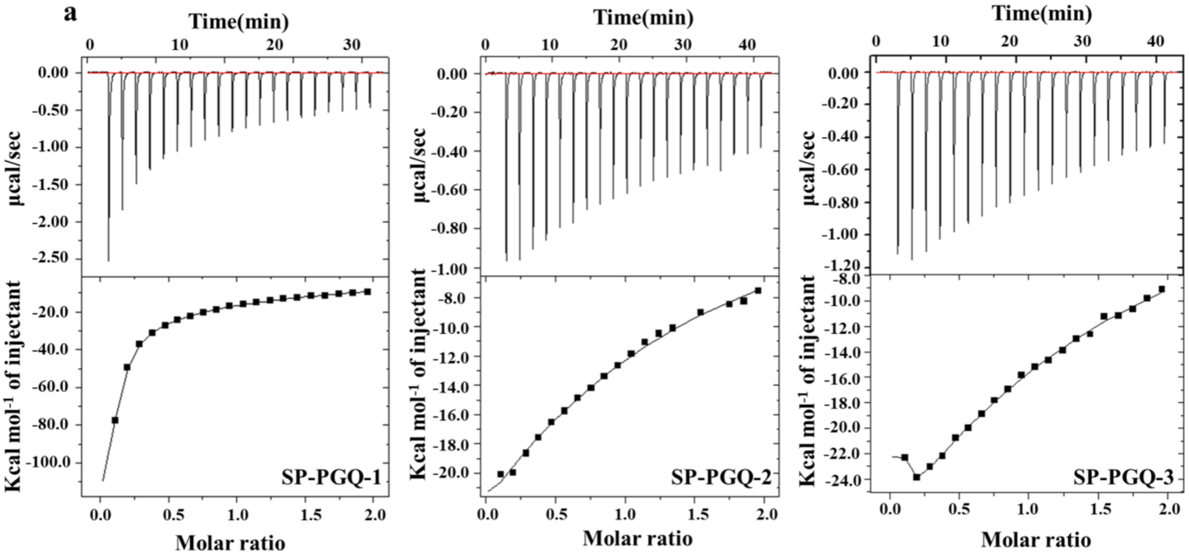

b

Thermodynamic parameters	SP-PGQ-1	SP-PGQ-2	SP-PGQ-3
**Observation from ITC analysis**			
ΔH1(cal/mol)	-2.696×10^5	-2.403×10^5	-6.009×10^4
K_a1 (M^{-1})	5.69×10^5	2.16×10^4	2.95×10^4
ΔH2 (cal/mol)	-6.272×10^6	-1.308×10^5	-2.223×10^4
K_a2 (M^{-1})	1.74×10^4	2.34×10^4	1.47×10^8

Supplementary Figure S4. Interaction of SP-PGQs with TMPyP2. (a) ITC thermograms of SP-PGQs with TMPyP2(a known non-G-quadruplex binder used as a negative control) and (**b)** List of thermodynamic parameters observed in ITC analysis.

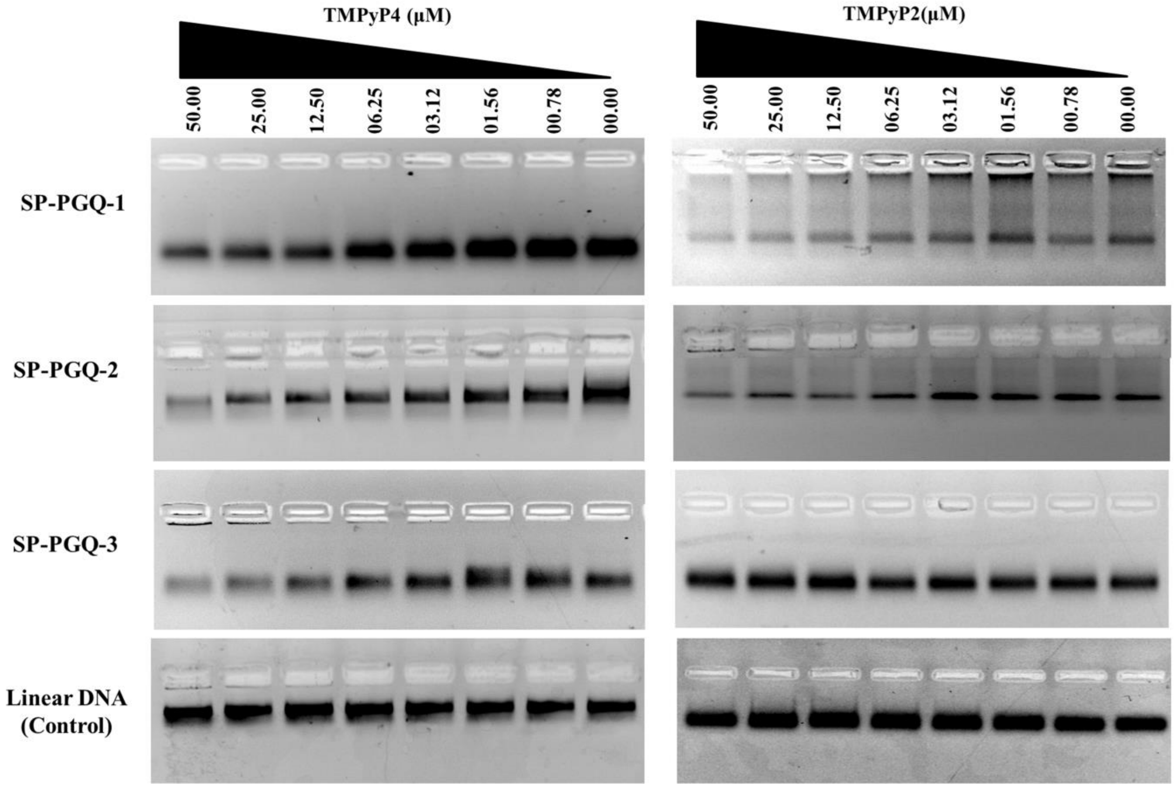

Supplementary Figure S5: Effect of TMPyP4 and TMPyP2 on the G-quadruplex formation in SP-PGQs. Effect of TMPyP4 and TMPyP2 on the Taq polymerase PCR stop assay of SP-PGQ-1, SP-PGQ-2, SP-PGQ-3 and Linear DNA(control).

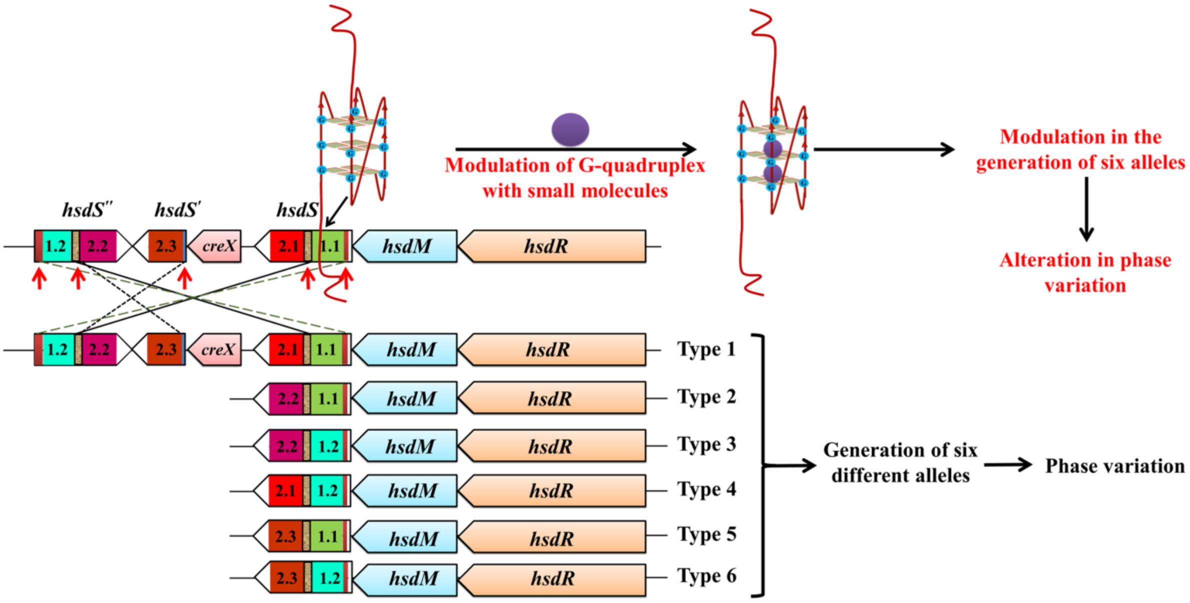

Supplementary Figure S6. Schematic representation of Type I Restriction-Modification system of *Streptococcus pneumoniae*. Type I RM system consists of *SpnD39III* locus which includes *hsdR*, *hsdM*, *creX* recombinase and three alleles of *hsdS*, one complete and two truncated(*hsdS´*, *hsdS´´*). Inverted repeats(depicted by the red arrow) leads to six different variations in *hsdS* protein. The G-quadruplex motif is present in *hsdS* gene region.(represented by the black arrow).

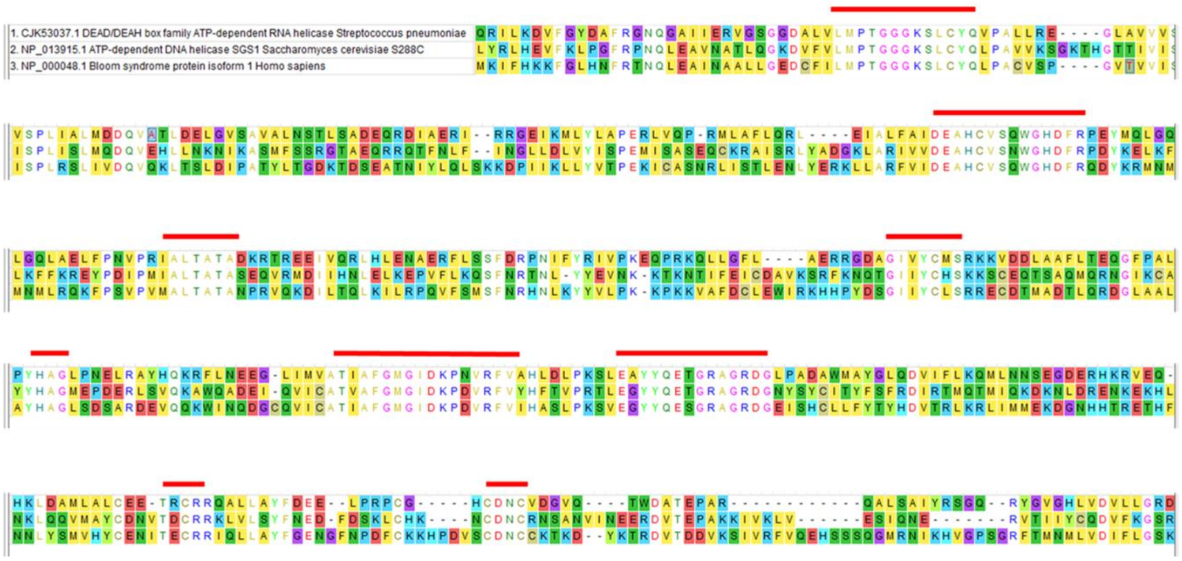

Supplementary Figure S7. RecQ helicases (Sgs1 of yeast and BLM of Human) homologs in *Streptococcus pneumoniae*: Multiple sequence alignment of Sgs1 (NP_013915-1), BLM (NP_000048-1) and there homolog DEAD RNA helicase (NP_358929-1) of *Streptococcus pneumoniae* constructed using Muscle algorithm in MEGA tool. (Similar motifs are depicted in red)

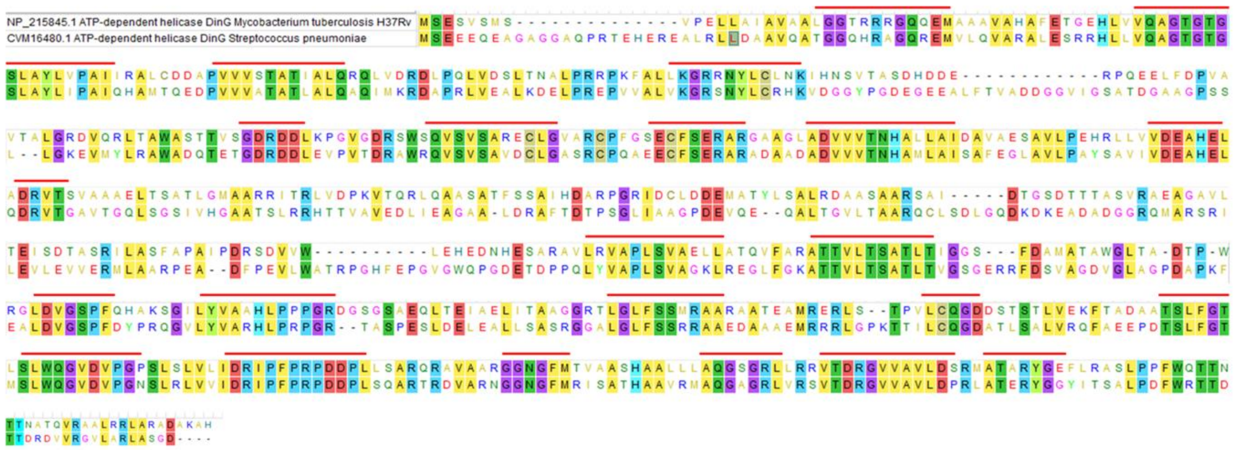

Supplementary Figure S8. Sequence and motif similarity between DinG of *Mycobacterium tuberculosis* and *Streptococcus pneumoniae*: Sequence alignment of DinG (NP_215845-1) of *Mycobacterium tuberculosis* and DinG(NP_358305-1) of *Streptococcus pneumoniae* constructed using Muscle algorithm in MEGA tool. (Similar motifs are depicted in red)

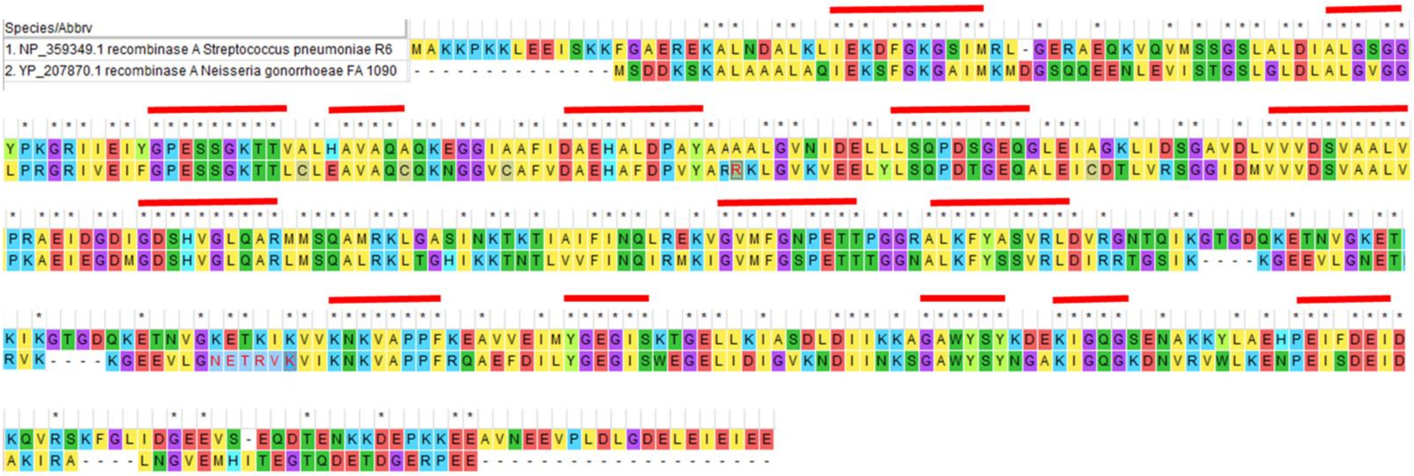

Supplementary Figure S9. Sequence similarity between RecA of *Neisseria gonorrhoea* and *Streptococcus pneumoniae*: Sequence alignment of RecA (NP_417179-1) **of *Escherichia coli*** and recombinase A (NP_359349-1) of *Streptococcus pneumoniae* constructed using Muscle algorithm in MEGA tool.

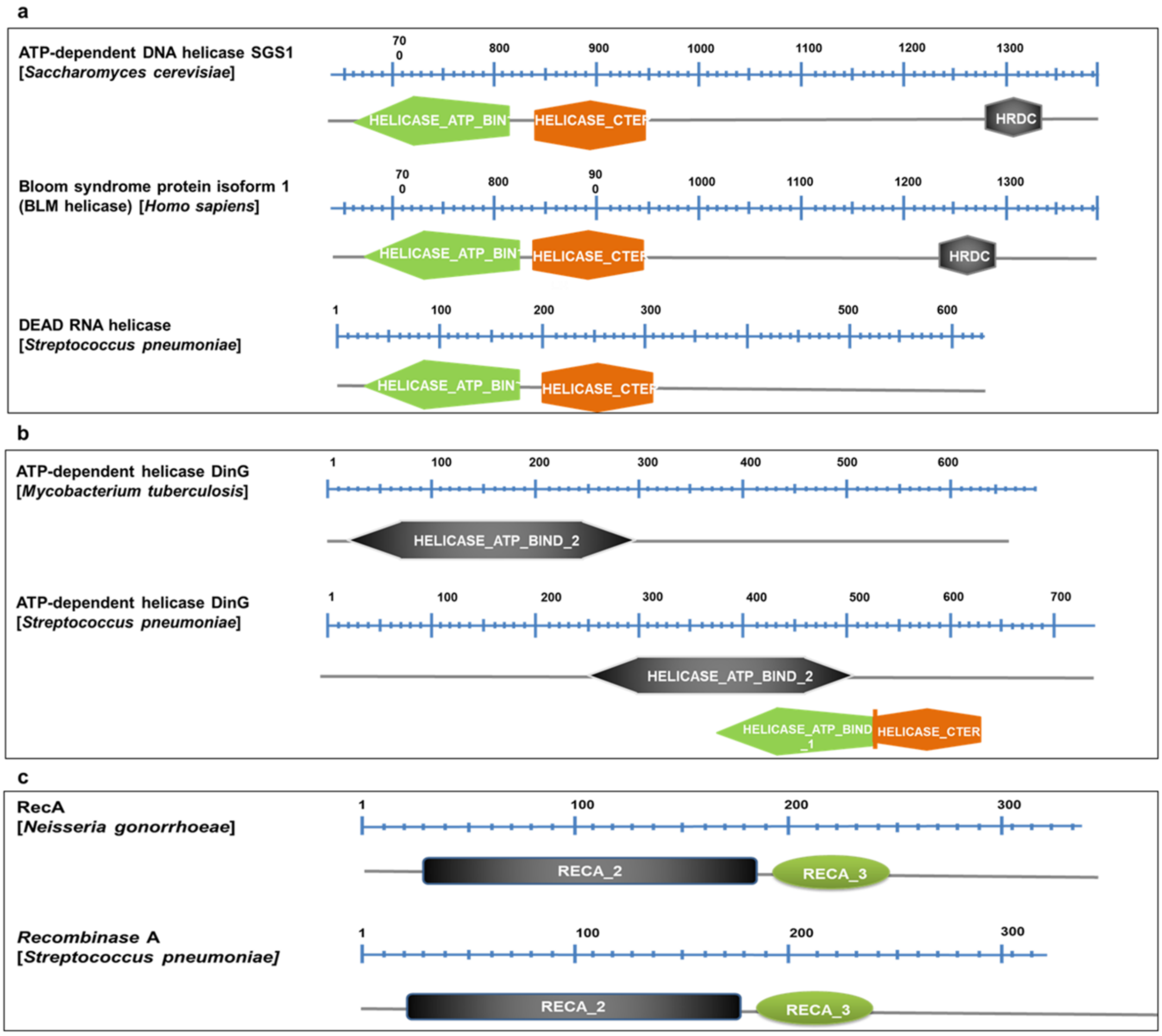

Supplementary Figure S10 Motif analysis using ScanProsite. (a) Conserved motifs prediction of Sgs1(Yeast), BLM helicase(Human) and DEAD RNA helicase(*Streptococcus pneumoniae*). All the three sequences preserve two conserved domains Helicase_ATP_Binding and Helicase_CTER. Sgs1 and BLM1 have an additional HRDC domain. (b) Conserved motifs prediction for DinG (*Mycobacterium tuberculosis*) and DinG(*Streptococcus pneumoniae*). Both the sequences possess a conserved domain - Helicase_ATP_Binding_2. In addition to this, *Streptococcus pneumoniae* also possess two additional domains – Helicase_ATP_Binding_1 and Helicase_CTER (c) Conserved motifs prediction for of recA of *Neisseria gonorrhheae* and recombinase A of *Streptococcus pneumoniae* showed both the sequences possess two conserved domains – RecA_2 and recA_3.

Supplementary Table S1: List of completely sequenced strains of *Streptococcus pneumoniae* taken for this study.

Strain Name	Genome Size (Mb)	GC%	Accession Number	Genes	Proteins
Streptococcus pneumoniae R6	2.03862	39.7	NC_003098.1	1967	1814
Streptococcus pneumoniae TIGR4	2.16084	39.7	NC_003028.3	2344	2092
Streptococcus pneumoniae D39	2.04611	39.7	NC_008533.1	2220	1996
Streptococcus pneumoniae 70585	2.18468	39.7	NC_012468.1	2397	2134
Streptococcus pneumoniae JJA	2.12023	39.7	NC_012466.1	2285	2057
Streptococcus pneumoniae P1031	2.11188	39.7	NC_012467.1	2336	2081
Streptococcus pneumoniae Taiwan19F-14	2.11215	39.8	NC_012469.1	2306	2052
Streptococcus pneumoniae Hungary19A-6	2.24561	39.6	NC_010380.1	2429	2167
Streptococcus pneumoniae G54	2.07895	39.7	NC_011072.1	2269	1986
Streptococcus pneumoniae CGSP14	2.2092	39.5	NC_010582.1	2380	2112
Streptococcus pneumoniae ATCC 700669	2.22132	39.5	NC_011900.1	2403	2150
Streptococcus pneumoniae AP200	2.13058	39.5	NC_014494.1	2291	2016
Streptococcus pneumoniae 670-6B	2.24004	39.6	NC_014498.1	2472	2199
Streptococcus pneumoniae TCH8431/19A	2.08877	39.8	NC_014251.1	2277	2031
Streptococcus pneumoniae INV200	2.09332	39.6	NC_017593.1	2264	2006
Streptococcus pneumoniae OXC141	2.03687	39.8	NC_017592.1	2246	2005
Streptococcus pneumoniae INV104	2.14212	39.6	NC_017591.1	2347	2065
Streptococcus pneumoniae SPN034156	2.02448	39.9	NC_021006.1	2220	1963
Streptococcus pneumoniae SPN034183	2.03725	39.8	NC_021028.1	2244	1986
Streptococcus pneumoniae SPN994038	2.02624	39.8	NC_021026.1	2232	1990
Streptococcus pneumoniae SPN994039	2.0265	39.8	NC_021005.1	2233	1992
Streptococcus pneumoniae SPN032672	2.13119	39.6	NC_021003.1	2362	2069
Streptococcus pneumoniae SPN033038	2.1335	39.6	NC_021004.1	2365	2063
Streptococcus pneumoniae ST556	2.15081	39.7	NC_017769.2	2359	2110
Streptococcus pneumoniae gamPNI0373	2.06415	39.8	NC_018630.1	2277	2026
Streptococcus pneumoniae NT_110_58	2.28777	39.8	NZ_CP007593.1	2445	2084
Streptococcus pneumoniae A66	1.98341	39.7	NZ_LN847353.1	2178	1934
Streptococcus pneumoniae NCTC7465	2.11097	39.7	NZ_LN831051.1	2330	2064
Streptococcus pneumoniae SP49	2.20664	39.9	NZ_CP018136.1	2415	2169
Streptococcus pneumoniae SP61	2.07181	39.9	NZ_CP018137.1	2245	2008
Streptococcus pneumoniae SP64	2.07311	39.9	NZ_CP018138.1	2249	2007
Streptococcus pneumoniae SWU02	2.09215	39.8	NZ_CP018347.1	2272	2019
Streptococcus pneumoniae KK0981	2.14848	39.6	NZ_AP017971.1	2319	2073
Streptococcus pneumoniae 19F	2.12552	39.6	CP025076.1	2276	2005
Streptococcus pneumoniae 11A	2.07919	39.8	CP018838.1	2317	2075
Streptococcus pneumoniae Xen35	2.16855	39.7	NZ_CP025256.1	2322	2052
Streptococcus pneumoniae 335	2.22132	39.5	CP026670.1	2375	2132
Streptococcus pneumoniae D39V	2.04657	39.7	CP027540.1	2151	1877
Streptococcus pneumoniae SPNA45	2.12993	39.8	HE983624.1	2083	1926

Supplementary Table S2: G-quadruplex prediction of the three most conserved PGQs of *Streptococcus pneumoniae* using QGRS Mapper tool.

#PGQ	Length	G4 Sequence	G-Score
SP- PGQ-1	36	GGGCAACTTGGCTGGGGTCTAGTTCCACGGGACGGG	63
SP- PGQ-2	22	GGGCTAGTGGGGGGAGGG	68
SP- PGQ-3	27	GGGCTAATAGGGAGAGCAGGGACGGGG	69

Supplementary Table S3: G-quadruplex prediction of the three most conserved PGQs of *Streptococcus pneumoniae* using PQSFinder tool.

#PGQ	Width	G4 Sequence	Score	Strand	Nt#	Nb$
SP- PGQ-1	36	GGGCAACTTGGCTGGGGTCTAGTTCCACGGGACGGG	45	+	3	0
SP- PGQ-2	22	GGGCTAGTGGGGGGAGGG	69	+	3	0
SP- PGQ-3	27	GGGCTAATAGGGAGAGCAGGGACGGGG	57	+	3	0

# Number of G-tracts; $ Number of bulges between G-tract;

Supplementary Table S4: The three conserved SP-PGQ's in various strains of *Streptococcus pneumoniae* along with the location in the respective strain's genomes and strand in which G-quadruplex is present predicted by our G-quadruplex prediction tool.

SP-PGQ-1								
Strain Accession Number	Length	Start Position	End Position	Strand	Strain Name	G-quadruplex Sequence	cG Score	cC Score
CP027540.1	36	370832	370867	>>>>	*Streptococcus pneumoniae* D39V	GGGCAACTTGGCTGGGGTCTAGTTCCACGGGACGGG	160	80
CP025256.1	36	381382	381417	>>>>	*Streptococcus pneumoniae* Xen35	GGGCAACTTGGCTGGGGTCTAGTTCCACGGGACGGG	160	80
AP018391.1	36	358052	358087	>>>>	*Streptococcus pneumoniae* MDRSPN001 DNA	GGGCAACTTGGCTGGGGTCTAGTTCCACGGGACGGG	160	80
AP017971.1	36	365066	365101	>>>>	*Streptococcus pneumoniae* DNA : KK0981	GGGCAACTTGGCTGGGGTCTAGTTCCACGGGACGGG	160	80
CP018347.1	36	799471	799506	>>>>	*Streptococcus pneumoniae* SWU02	GGGCAACTTGGCTGGGGTCTAGTTCCACGGGACGGG	160	80
CP018138.1	36	368819	368854	>>>>	*Streptococcus pneumoniae* SP64	GGGCAACTTGGCTGGGGTCTAGTTCCACGGGACGGG	160	80
CP018137.1	36	367157	367192	>>>>	*Streptococcus pneumoniae* SP61	GGGCAACTTGGCTGGGGTCTAGTTCCACGGGACGGG	160	80
CP018136.1	36	445409	445444	>>>>	*Streptococcus pneumoniae* SP49	GGGCAACTTGGCTGGGGTCTAGTTCCACGGGACGGG	160	80
CP016227.1	36	404664	404699	>>>>	*Streptococcus pneumoniae* D219	GGGCAACTTGGCTGGGGTCTAGTTCCACGGGACGGG	160	80
CP003357.2	36	445691	445726	>>>>	*Streptococcus pneumoniae* ST556	GGGCAACTTGGCTGGGGTCTAGTTCCACGGGACGGG	160	80
LN847353.1	36	366432	366467	>>>>	*Streptococcus pneumoniae* _A66_v1	GGGCAACTTGGCTGGGGTCTAGTTCCACGGGACGGG	160	80
LN831051.1	36	102726	102691	<<<<	*Streptococcus pneumoniae* NCTC7465	GGGCAACTTGGCTGGGGTCTAGTTCCACGGGACGGG	160	80
CP007593.1	36	360684	360719	>>>>	*Streptococcus pneumoniae* NT_110_58	GGGCAACTTGGCTGGGGTCTAGTTCCACGGGACGGG	160	80
FQ312041.2	36	377847	377882	>>>>	*Streptococcus pneumoniae* SPN994038	GGGCAACTTGGCTGGGGTCTAGTTCCACGGGACGGG	160	80
FQ312044.2	36	377847	377882	>>>>	*Streptococcus pneumoniae* SPN994039	GGGCAACTTGGCTGGGGTCTAGTTCCACGGGACGGG	160	80
CP001845.1	36	395949	395984	>>>>	*Streptococcus pneumoniae* gamPNI0373	GGGCAACTTGGCTGGGGTCTAGTTCCACGGGACGGG	160	80
HE983624.1	36	1676391	1676356	>>>>	*Streptococcus pneumoniae* SPNA45	GGGCAACTTGGCTGGGGTCTAGTTCCACGGGACGGG	160	80
CP002176.1	36	432084	432119	>>>>	*Streptococcus*	GGGCAACTTGGCTGGGGTCTAGTTCCACGGGACGGG	160	80

					pneumoniae 670-6B			
CP002121.1	36	391339	391374	>>>>	*Streptococcus pneumoniae* AP200	GGGCAACTTGGCTGGGGTCTAGTTCCACGGGACGGG	160	80
FQ312029.1	36	369110	369145	>>>>	*Streptococcus pneumoniae* INV200	GGGCAACTTGGCTGGGGTCTAGTTCCACGGGACGGG	160	80
FQ312027.1	36	388566	388601	>>>>	*Streptococcus pneumoniae* OXC141	GGGCAACTTGGCTGGGGTCTAGTTCCACGGGACGGG	160	80
FQ312045.1	36	1488451	1488486	>>>>	*Streptococcus pneumoniae* SPN034156	GGGCAACTTGGCTGGGGTCTAGTTCCACGGGACGGG	160	80
FQ312043.1	36	388270	388305	>>>>	*Streptococcus pneumoniae* SPN034183	GGGCAACTTGGCTGGGGTCTAGTTCCACGGGACGGG	160	80
FQ312042.1	36	1547536	1547571	>>>>	*Streptococcus pneumoniae* SPN033038	GGGCAACTTGGCTGGGGTCTAGTTCCACGGGACGGG	160	80
FQ312039.1	36	1546220	1546255	>>>>	*Streptococcus pneumoniae* SPN032672	GGGCAACTTGGCTGGGGTCTAGTTCCACGGGACGGG	160	80
FQ312030.1	36	387112	387147	>>>>	*Streptococcus pneumoniae* INV104	GGGCAACTTGGCTGGGGTCTAGTTCCACGGGACGGG	160	80
CP001993.1	36	631969	632004	>>>>	*Streptococcus pneumoniae* TCH8431/19A	GGGCAACTTGGCTGGGGTCTAGTTCCACGGGACGGG	160	80
CP000921.1	36	406696	406731	>>>>	*Streptococcus pneumoniae* Taiwan19F-14	GGGCAACTTGGCTGGGGTCTAGTTCCACGGGACGGG	160	80
CP000920.1	36	387407	387442	>>>>	*Streptococcus pneumoniae* P1031	GGGCAACTTGGCTGGGGTCTAGTTCCACGGGACGGG	160	80
CP000919.1	36	375149	375184	>>>>	*Streptococcus pneumoniae* JJA	GGGCAACTTGGCTGGGGTCTAGTTCCACGGGACGGG	160	80
CP000918.1	36	425822	425857	>>>>	*Streptococcus pneumoniae* 70585	GGGCAACTTGGCTGGGGTCTAGTTCCACGGGACGGG	160	80
AE007317.1	36	362846	362881	>>>>	*Streptococcus pneumoniae* R6	GGGCAACTTGGCTGGGGTCTAGTTCCACGGGACGGG	160	80
AE005672.3	36	381383	381418	>>>>	*Streptococcus pneumoniae* TIGR4	GGGCAACTTGGCTGGGGTCTAGTTCCACGGGACGGG	160	80
CP001033.1	36	396618	396653	>>>>	*Streptococcus pneumoniae* CGSP14	GGGCAACTTGGCTGGGGTCTAGTTCCACGGGACGGG	160	80
CP000936.1	36	463762	463797	>>>>	*Streptococcus pneumoniae* Hungary19A-6	GGGCAACTTGGCTGGGGTCTAGTTCCACGGGACGGG	160	80
CP000410.1	36	370375	370410	>>>>	*Streptococcus pneumoniae* D39	GGGCAACTTGGCTGGGGTCTAGTTCCACGGGACGGG	160	80
**SP-PGQ-2**								
AP018391.1	24	1123986	1124005	>>>>	*Streptococcus pneumoniae* MDRSPN001 DNA	GGGCTAGTGGGGGGAGGGGG	150	10
AP018391.1	24	1126328	1126347	>>>>	*Streptococcus pneumoniae*	GGGCTAGTGGGGGGAGGGGG	150	10

					**SWU02**			
**CP018347.1**	**24**	**1641338**	**1641357**	**>>>>**	***Streptococcus pneumoniae*** **SP64**	**GGGCTAGTGGGGGGAGGGGG**	**150**	**10**
**CP018138.1**	**24**	**1210671**	**1210690**	**>>>>**	***Streptococcus pneumoniae*** **SP61**	**GGGCTAGTGGGGGGAGGGGG**	**150**	**10**
**CP018137.1**	**24**	**1208971**	**1208990**	**>>>>**	***Streptococcus pneumoniae*** **ST556**	**GGGCTAGTGGGGGGAGGGGG**	**150**	**10**
**CP003357.2**	**24**	**1288739**	**1288758**	**>>>>**	***Streptococcus pneumoniae*** **NCTC7465**	**GGGCTAGTGGGGGGAGGGGG**	**150**	**10**
**LN831051.1**	**24**	**1770757**	**1770776**	**>>>>**	***Streptococcus pneumoniae*** **NT_110_58**	**GGGCTAGTGGGGGGAGGGGG**	**150**	**10**
**CP006844.1**	**24**	**1207721**	**1207740**	**>>>>**	***Streptococcus pneumoniae*** **670-6B**	**GGGCTAGTGGGGGGAGGGGG**	**150**	**10**
**CP002176.1**	**24**	**1344921**	**1344940**	**>>>>**	***Streptococcus pneumoniae*** **TCH8431/19A**	**GGGCTAGTGGGGGGAGGGGG**	**150**	**10**
**CP001993.1**	**24**	**1473873**	**1473892**	**>>>>**	***Streptococcus pneumoniae*** **Taiwan19F-14**	**GGGCTAGTGGGGGGAGGGGG**	**150**	**10**
**CP000921.1**	**24**	**1250122**	**1250141**	**>>>>**	***Streptococcus pneumoniae*** **Xen35**	**GGGCTAGTGGGGGGAGGGGG**	**150**	**10**
**CP025256.1**	**24**	**837583**	**837564**	**<<<<**	***Streptococcus pneumoniae*** **11A**	**GGGCTAGTGGGGGGAGGGGG**	**150**	**10**
**CP018838.1**	**24**	**355906**	**355887**	**<<<<**	***Streptococcus pneumoniae*** **19F**	**GGGCTAGTGGGGGGAGGGGG**	**150**	**10**
**CP025076.1**	**24**	**7466**	**7447**	**<<<<**	***Streptococcus pneumoniae*** **DNA nearly : KK1157**	**GGGCTAGTGGGGGGAGGGGG**	**150**	**10**
**CP016632.2**	**24**	**825609**	**825590**	**<<<<**	***Streptococcus pneumoniae*** **DNA : KK0981**	**GGGCTAGTGGGGGGAGGGGG**	**150**	**10**
**AP017971.1**	**24**	**812706**	**812687**	**<<<<**	***Streptococcus pneumoniae*** **SP49**	**GGGCTAGTGGGGGGAGGGGG**	**150**	**10**
**KM030255.1**	**24**	**2813**	**2794**	**<<<<**	***Streptococcus pneumoniae*** **gamPNI0373**	**GGGCTAGTGGGGGGAGGGGG**	**150**	**10**
**CP001845.1**	**24**	**826932**	**826913**	**<<<<**	***Streptococcus pneumoniae*** **AP200**	**GGGCTAGTGGGGGGAGGGGG**	**150**	**10**
**CP002121.1**	**24**	**875524**	**875505**	**<<<<**	***Streptococcus pneumoniae*** **INV200**	**GGGCTAGTGGGGGGAGGGGG**	**150**	**10**
**FQ312029.1**	**24**	**801135**	**801116**	**<<<<**	***Streptococcus pneumoniae*** **SPN033038**	**GGGCTAGTGGGGGGAGGGGG**	**150**	**10**
**FQ312042.1**	**24**	**1985351**	**1985332**	**<<<<**	***Streptococcus pneumoniae*** **SPN032672**	**GGGCTAGTGGGGGGAGGGGG**	**150**	**10**
**FQ312039.1**	**24**	**1983177**	**1983158**	**<<<<**	***Streptococcus pneumoniae*** **INV104**	**GGGCTAGTGGGGGGAGGGGG**	**150**	**10**
**FQ312030.1**	**24**	**821970**	**821951**	**<<<<**	***Streptococcus pneumoniae***	**GGGCTAGTGGGGGGAGGGGG**	**150**	**10**

					P1031			
CP000920.1	24	817097	817078	<<<<	*Streptococcus pneumoniae* JJA	GGGCTAGTGGGGGGAGGGGG	150	10
CP000919.1	24	796656	796637	<<<<	*Streptococcus pneumoniae* 70585	GGGCTAGTGGGGGGAGGGGG	150	10
CP000918.1	24	850550	850531	<<<<	*Streptococcus pneumoniae* G54	GGGCTAGTGGGGGGAGGGGG	150	10
CP001015.1	24	787737	787718	<<<<	*Streptococcus pneumoniae* TIGR4	GGGCTAGTGGGGGGAGGGGG	150	10
AE005672.3	24	837583	837564	<<<<	*Streptococcus pneumoniae* CGSP14	GGGCTAGTGGGGGGAGGGGG	150	10
CP001033.1	24	825702	825683	<<<<	*Streptococcus pneumoniae* Hungary19A-6	GGGCTAGTGGGGGGAGGGGG	150	10
CP000936.1	24	918307	918288	<<<<	*Streptococcus pneumoniae* 335	GGGCTAGTGGGGGGAGGGGG	150	10
AP018043.1	24	348898	348879	<<<<	*Streptococcus pneumoniae* SPN994038	GGGCTAGTGGGGGGAGGGGG	150	10
FQ312041.2	24	797468	797449	<<<<	*Streptococcus pneumoniae* SPN994039	GGGCTAGTGGGGGGAGGGGG	150	10
FQ312044.2	24	797468	797449	<<<<	*Streptococcus pneumoniae* OXC141	GGGCTAGTGGGGGGAGGGGG	150	10
FQ312027.1	24	807849	807830	<<<<	*Streptococcus pneumoniae* SPN034156	GGGCTAGTGGGGGGAGGGGG	150	10
FQ312045.1	24	1912022	1912003	<<<<	*Streptococcus pneumoniae* SPN034183	GGGCTAGTGGGGGGAGGGGG	150	10
FQ312043.1	24	807948	807929	<<<<	*Streptococcus pneumoniae* D39V	GGGCTAGTGGGGGGAGGGGG	150	10
**SP-PGQ-3**								
CP026670.1	27	341866	341892	>>>>	*Streptococcus pneumoniae* 335	GGGCTAATAGGGAGAGCAGGGACGGGG	150	30
CP025256.1	27	358212	358238	>>>>	*Streptococcus pneumoniae* Xen35	GGGCTAATAGGGAGAGCAGGGACGGGG	150	30
CP018838.1	27	1972727	1972753	>>>>	*Streptococcus pneumoniae* 11A	GGGCTAATAGGGAGAGCAGGGACGGGG	150	30
CP025076.1	27	1666804	1666830	>>>>	*Streptococcus pneumoniae* 19F	GGGCTAATAGGGAGAGCAGGGACGGGG	150	30
AP018391.1	27	343145	343171	>>>>	*Streptococcus pneumoniae* MDRSPN001 DNA	GGGCTAATAGGGAGAGCAGGGACGGGG	150	30
AP017971.1	27	348924	348950	>>>>	*Streptococcus pneumoniae* DNA : KK0981	GGGCTAATAGGGAGAGCAGGGACGGGG	150	30

CP018347.1	27	784564	784590	>>>>	*Streptococcus pneumoniae* SWU02	GGGCTAATAGGGAGAGCAGGGACGGGG	150	30
CP018138.1	27	353895	353921	>>>>	*Streptococcus pneumoniae* SP64	GGGCTAATAGGGAGAGCAGGGACGGGG	150	30
CP018137.1	27	352233	352259	>>>>	*Streptococcus pneumoniae* SP61	GGGCTAATAGGGAGAGCAGGGACGGGG	150	30
CP018136.1	27	430621	430647	>>>>	*Streptococcus pneumoniae* SP49	GGGCTAATAGGGAGAGCAGGGACGGGG	150	30
CP003357.2	27	430784	430810	>>>>	*Streptococcus pneumoniae* ST556	GGGCTAATAGGGAGAGCAGGGACGGGG	150	30
LN847353.1	27	344412	344438	>>>>	*Streptococcus pneumoniae* _A66_v1	GGGCTAATAGGGAGAGCAGGGACGGGG	150	30
LN831051.1	27	117629	117603	<<<<	*Streptococcus pneumoniae* NCTC7465	GGGCTAATAGGGAGAGCAGGGACGGGG	150	30
CP007593.1	27	345779	345805	>>>>	*Streptococcus pneumoniae* NT_110_58	GGGCTAATAGGGAGAGCAGGGACGGGG	150	30
FQ312041.2	27	362943	362969	>>>>	*Streptococcus pneumoniae* SPN994038	GGGCTAATAGGGAGAGCAGGGACGGGG	150	30
FQ312044.2	27	362943	362969	>>>>	*Streptococcus pneumoniae* SPN994039	GGGCTAATAGGGAGAGCAGGGACGGGG	150	30
CP001845.1	27	381044	381070	>>>>	*Streptococcus pneumoniae* gamPNI0373	GGGCTAATAGGGAGAGCAGGGACGGGG	150	30
HE983624.1	27	1699020	1698994	<<<<	*Streptococcus pneumoniae* SPNA45	GGGCTAATAGGGAGAGCAGGGACGGGG	150	30
CP002176.1	27	408899	408925	>>>>	*Streptococcus pneumoniae* 670-6B	GGGCTAATAGGGAGAGCAGGGACGGGG	150	30
CP002121.1	27	376436	376462	>>>>	*Streptococcus pneumoniae* AP200	GGGCTAATAGGGAGAGCAGGGACGGGG	150	30
FQ312029.1	27	346042	346068	>>>>	*Streptococcus pneumoniae* INV200	GGGCTAATAGGGAGAGCAGGGACGGGG	150	30
FQ312027.1	27	373662	373688	>>>>	*Streptococcus pneumoniae* OXC141	GGGCTAATAGGGAGAGCAGGGACGGGG	150	30
FQ312045.1	27	1473546	1473572	>>>>	*Streptococcus pneumoniae* SPN034156	GGGCTAATAGGGAGAGCAGGGACGGGG	150	30
FQ312043.1	27	373366	373392	>>>>	*Streptococcus pneumoniae* SPN034183	GGGCTAATAGGGAGAGCAGGGACGGGG	150	30
FQ312042.1	27	1532636	1532662	>>>>	*Streptococcus pneumoniae* SPN033038	GGGCTAATAGGGAGAGCAGGGACGGGG	150	30
FQ312039.1	27	1531320	1531346	>>>>	*Streptococcus pneumoniae* SPN032672	GGGCTAATAGGGAGAGCAGGGACGGGG	150	30
FQ312030.1	27	364176	364202	>>>>	*Streptococcus pneumoniae*	GGGCTAATAGGGAGAGCAGGGACGGGG	150	30

					INV104			
CP001993.1	27	617045	617071	>>>>	*Streptococcus pneumoniae* TCH8431/19A	GGGCTAATAGGGAGAGCAGGGACGGGG	150	30
CP000921.1	27	391789	391815	>>>>	*Streptococcus pneumoniae* Taiwan19F-14	GGGCTAATAGGGAGAGCAGGGACGGGG	150	30
CP000920.1	27	372502	372528	>>>>	*Streptococcus pneumoniae* P1031	GGGCTAATAGGGAGAGCAGGGACGGGG	150	30
CP000919.1	27	351972	351998	>>>>	*Streptococcus pneumoniae* JJA	GGGCTAATAGGGAGAGCAGGGACGGGG	150	30
CP000918.1	27	402741	402767	>>>>	*Streptococcus pneumoniae* 70585	GGGCTAATAGGGAGAGCAGGGACGGGG	150	30
FM211187.1	27	341866	341892	>>>>	*Streptococcus pneumoniae* ATCC 700669	GGGCTAATAGGGAGAGCAGGGACGGGG	150	30
CP001015.1	27	338145	338171	>>>>	*Streptococcus pneumoniae* G54	GGGCTAATAGGGAGAGCAGGGACGGGG	150	30
AE005672.3	27	358213	358239	>>>>	*Streptococcus pneumoniae* TIGR4	GGGCTAATAGGGAGAGCAGGGACGGGG	150	30
CP001033.1	27	373576	373602	>>>>	*Streptococcus pneumoniae* CGSP14	GGGCTAATAGGGAGAGCAGGGACGGGG	150	30
CP000936.1	27	440677	440703	>>>>	*Streptococcus pneumoniae* Hungary19A-6	GGGCTAATAGGGAGAGCAGGGACGGGG	150	30

Supplementary Table S5: Multiple sequence alignment of SP-PGQ-1

Consensus	TTCTTCGATCAGGTAGGAGAGCTCTTGGGCAACTTGGCTGGGGTCTAGTTCCACGGGACGGGAAGACTCAAGGAGAGTAAGGGTTTG
CP027540.1	TTCTTCGATCAGGTAGGAGAGCTCTTGGGCAACTTGGCTGGGGTCTAGTTCCACGGGACGGGAAGACTCAAGGAGAGTAAGGGTTTG
CP025256.1	TTCTTCGATCAGGTAGGAGAGCTCTTGGGCAACTTGGCTGGGGTCTAGTTCCACGGGACGGGAAGACTCAAGGAGAGTAAGGGTTTG
AP018391.1	TTCTTCGATCAGGTAGGAGAGCTCTTGGGCAACTTGGCTGGGGTCTAGTTCCACGGGACGGGAAGACTCAAGGAGAGTAAGGGTTTG
AP017971.1	TTCTTCGATCAGGTAGGAGAGCTCTTGGGCAACTTGGCTGGGGTCTAGTTCCACGGGACGGGAAGACTCAAGGAGAGTAAGGGTTTG
CP018347.1	TTCTTCGATCAGGTAGGAGAGCTCTTGGGCAACTTGGCTGGGGTCTAGTTCCACGGGACGGGAAGACTCAAGGAGAGTAAGGGTTTG
CP018138.1	TTCTTCGATCAGGTAGGAGAGCTCTTGGGCAACTTGGCTGGGGTCTAGTTCCACGGGACGGGAAGACTCAAGGAGAGTAAGGGTTTG
CP018137.1	TTCTTCGATCAGGTAGGAGAGCTCTTGGGCAACTTGGCTGGGGTCTAGTTCCACGGGACGGGAAGACTCAAGGAGAGTAAGGGTTTG
CP018136.1	TTCTTCGATCAGGTAGGAGAGCTCTTGGGCAACTTGGCTGGGGTCTAGTTCCACGGGACGGGAAGACTCAAGGAGAGTAAGGGTTTG
CP016227.1	TTCTTCGATCAGGTAGGAGAGCTCTTGGGCAACTTGGCTGGGGTCTAGTTCCACGGGACGGGAAGACTCAAGGAGAGTAAGGGTTTG
CP003357.2	TTCTTCGATCAGGTAGGAGAGCTCTTGGGCAACTTGGCTGGGGTCTAGTTCCACGGGACGGGAAGACTCAAGGAGAGTAAGGGTTTG
LN847353.1	TTCTTCGATCAGGTAGGAGAGCTCTTGGGCAACTTGGCTGGGGTCTAGTTCCACGGGACGGGAAGACTCAAGGAGAGTAAGGGTTTG
LN831051.1	TTCTTCGATCAGGTAGGAGAGCTCTTGGGCAACTTGGCTGGGGTCTAGTTCCACGGGACGGGAAGACTCAAGGAGAGTAAGGGTTTG
CP007593.1	TTCTTCGATCAGGTAGGAGAGCTCTTGGGCAACTTGGCTGGGGTCTAGTTCCACGGGACGGGAAGACTCAAGGAGAGTAAGGGTTTG
FQ312041.2	TTCTTCGATCAGGTAGGAGAGCTCTTGGGCAACTTGGCTGGGGTCTAGTTCCACGGGACGGGAAGACTCAAGGAGAGTAAGGGTTTG
FQ312044.2	TTCTTCGATCAGGTAGGAGAGCTCTTGGGCAACTTGGCTGGGGTCTAGTTCCACGGGACGGGAAGACTCAAGGAGAGTAAGGGTTTG
CP001845.1	TTCTTCGATCAGGTAGGAGAGCTCTTGGGCAACTTGGCTGGGGTCTAGTTCCACGGGACGGGAAGACTCAAGGAGAGTAAGGGTTTG
HE983624.1	TTCTTCGATCAGGTAGGAGAGCTCTTGGGCAACTTGGCTGGGGTCTAGTTCCACGGGACGGGAAGACTCAAGGAGAGTAAGGGTTTG
CP002176.1	TTCTTCGATCAGGTAGGAGAGCTCTTGGGCAACTTGGCTGGGGTCTAGTTCCACGGGACGGGAAGACTCAAGGAGAGTAAGGGTTTG
CP002121.1	TTCTTCGATCAGGTAGGAGAGCTCTTGGGCAACTTGGCTGGGGTCTAGTTCCACGGGACGGGAAGACTCAAGGAGAGTAAGGGTTTG
FQ312029.1	TTCTTCGATCAGGTAGGAGAGCTCTTGGGCAACTTGGCTGGGGTCTAGTTCCACGGGACGGGAAGACTCAAGGAGAGTAAGGGTTTG
FQ312027.1	TTCTTCGATCAGGTAGGAGAGCTCTTGGGCAACTTGGCTGGGGTCTAGTTCCACGGGACGGGAAGACTCAAGGAGAGTAAGGGTTTG
FQ312045.1	TTCTTCGATCAGGTAGGAGAGCTCTTGGGCAACTTGGCTGGGGTCTAGTTCCACGGGACGGGAAGACTCAAGGAGAGTAAGGGTTTG
FQ312043.1	TTCTTCGATCAGGTAGGAGAGCTCTTGGGCAACTTGGCTGGGGTCTAGTTCCACGGGACGGGAAGACTCAAGGAGAGTAAGGGTTTG
FQ312042.1	TTCTTCGATCAGGTAGGAGAGCTCTTGGGCAACTTGGCTGGGGTCTAGTTCCACGGGACGGGAAGACTCAAGGAGAGTAAGGGTTTG
FQ312039.1	TTCTTCGATCAGGTAGGAGAGCTCTTGGGCAACTTGGCTGGGGTCTAGTTCCACGGGACGGGAAGACTCAAGGAGAGTAAGGGTTTG
FQ312030.1	TTCTTCGATCAGGTAGGAGAGCTCTTGGGCAACTTGGCTGGGGTCTAGTTCCACGGGACGGGAAGACTCAAGGAGAGTAAGGGTTTG
CP001993.1	TTCTTCGATCAGGTAGGAGAGCTCTTGGGCAACTTGGCTGGGGTCTAGTTCCACGGGACGGGAAGACTCAAGGAGAGTAAGGGTTTG
CP000921.1	TTCTTCGATCAGGTAGGAGAGCTCTTGGGCAACTTGGCTGGGGTCTAGTTCCACGGGACGGGAAGACTCAAGGAGAGTAAGGGTTTG
CP000920.1	TTCTTCGATCAGGTAGGAGAGCTCTTGGGCAACTTGGCTGGGGTCTAGTTCCACGGGACGGGAAGACTCAAGGAGAGTAAGGGTTTG
CP000919.1	TTCTTCGATCAGGTAGGAGAGCTCTTGGGCAACTTGGCTGGGGTCTAGTTCCACGGGACGGGAAGACTCAAGGAGAGTAAGGGTTTG
CP000918.1	TTCTTCGATCAGGTAGGAGAGCTCTTGGGCAACTTGGCTGGGGTCTAGTTCCACGGGACGGGAAGACTCAAGGAGAGTAAGGGTTTG
AE007317.1	TTCTTCGATCAGGTAGGAGAGCTCTTGGGCAACTTGGCTGGGGTCTAGTTCCACGGGACGGGAAGACTCAAGGAGAGTAAGGGTTTG
AE005672.3	TTCTTCGATCAGGTAGGAGAGCTCTTGGGCAACTTGGCTGGGGTCTAGTTCCACGGGACGGGAAGACTCAAGGAGAGTAAGGGTTTG
CP001033.1	TTCTTCGATCAGGTAGGAGAGCTCTTGGGCAACTTGGCTGGGGTCTAGTTCCACGGGACGGGAAGACTCAAGGAGAGTAAGGGTTTG
CP000936.1	TTCTTCGATCAGGTAGGAGAGCTCTTGGGCAACTTGGCTGGGGTCTAGTTCCACGGGACGGGAAGACTCAAGGAGAGTAAGGGTTTG
CP000410.1	TTCTTCGATCAGGTAGGAGAGCTCTTGGGCAACTTGGCTGGGGTCTAGTTCCACGGGACGGGAAGACTCAAGGAGAGTAAGGGTTTG

Supplementary Table S6: Multiple sequence alignment of SP-PGQ-2

Consensus	AGTCTGCGAACTCATTTGGGAGGGCTAGTGGGGGGAGGGGGATGGAAATATTTAATAAATC
AP018391.1	AGTCTGCGAACTCATTTGGGAGGGCTAGTGGGGGGAGGGGGATGGAAATATTTAATAAATC
AP018391.1	AGTCTGCGAACTCATTTGGGAGGGCTAGTGGGGGGAGGGGGATGGAAATATTTAATAAATC
CP018347.1	AGTCTGCGAACTCATTTGGGAGGGCTAGTGGGGGGAGGGGGATGGAAATATTTAATAAATC
CP018138.1	AGTCTGCGAACTCATTTGGGAGGGCTAGTGGGGGGAGGGGGATGGAAATATTTAATAAATC
CP018137.1	AGTCTGCGAACTCATTTGGGAGGGCTAGTGGGGGGAGGGGGATGGAAATATTTAATAAATC
CP003357.2	AGTCTGCGAACTCATTTGGGAGGGCTAGTGGGGGGAGGGGGATGGAAATATTTAATAAATC
LN831051.1	AGTCTGCGAACTCATTTGGGAGGGCTAGTGGGGGGAGGGGGATGGAAATATTTAATAAATC
CP006844.1	AGTCTGCGAACTCATTTGGGAGGGCTAGTGGGGGGAGGGGGATGGAAATATTTAATAAATC
CP002176.1	AGTCTGCGAACTCATTTGGGAGGGCTAGTGGGGGGAGGGGGATGGAAATATTTAATAAATC
CP001993.1	AGTCTGCGAACTCATTTGGGAGGGCTAGTGGGGGGAGGGGGATGGAAATATTTAATAAATC
CP000921.1	AGTCTGCGAACTCATTTGGGAGGGCTAGTGGGGGGAGGGGGATGGAAATATTTAATAAATC
CP025256.1	AGTCTGCGAACTCATTTGGGAGGGCTAGTGGGGGGAGGGGGATGGAAATATTTAATAAATC
CP018838.1	AGTCTGCGAACTCATTTGGGAGGGCTAGTGGGGGGAGGGGGATGGAAATATTTAATAAATC
CP025076.1	AGTCTGCGAACTCATTTGGGAGGGCTAGTGGGGGGAGGGGGATGGAAATATTTAATAAATC
CP016632.2	AGTCTGCGAACTCATTTGGGAGGGCTAGTGGGGGGAGGGGGATGGAAATATTTAATAAATC
AP017971.1	AGTCTGCGAACTCATTTGGGAGGGCTAGTGGGGGGAGGGGGATGGAAATATTTAATAAATC
KM030255.1	AGTCTGCGAACTCATTTGGGAGGGCTAGTGGGGGGAGGGGGATGGAAATATTTAATAAATC
CP001845.1	AGTCTGCGAACTCATTTGGGAGGGCTAGTGGGGGGAGGGGGATGGAAATATTTAATAAATC
CP002121.1	AGTCTGCGAACTCATTTGGGAGGGCTAGTGGGGGGAGGGGGATGGAAATATTTAATAAATC
FQ312029.1	AGTCTGCGAACTCATTTGGGAGGGCTAGTGGGGGGAGGGGGATGGAAATATTTAATAAATC
FQ312042.1	AGTCTGCGAACTCATTTGGGAGGGCTAGTGGGGGGAGGGGGATGGAAATATTTAATAAATC
FQ312039.1	AGTCTGCGAACTCATTTGGGAGGGCTAGTGGGGGGAGGGGGATGGAAATATTTAATAAATC
FQ312030.1	AGTCTGCGAACTCATTTGGGAGGGCTAGTGGGGGGAGGGGGATGGAAATATTTAATAAATC
CP000920.1	AGTCTGCGAACTCATTTGGGAGGGCTAGTGGGGGGAGGGGGATGGAAATATTTAATAAATC
CP000919.1	AGTCTGCGAACTCATTTGGGAGGGCTAGTGGGGGGAGGGGGATGGAAATATTTAATAAATC
CP000918.1	AGTCTGCGAACTCATTTGGGAGGGCTAGTGGGGGGAGGGGGATGGAAATATTTAATAAATC
CP001015.1	AGTCTGCGAACTCATTTGGGAGGGCTAGTGGGGGGAGGGGGATGGAAATATTTAATAAATC
AE005672.3	AGTCTGCGAACTCATTTGGGAGGGCTAGTGGGGGGAGGGGGATGGAAATATTTAATAAATC
CP001033.1	AGTCTGCGAACTCATTTGGGAGGGCTAGTGGGGGGAGGGGGATGGAAATATTTAATAAATC
CP000936.1	AGTCTGCGAACTCATTTGGGAGGGCTAGTGGGGGGAGGGGGATGGAAATATTTAATAAATC
AP018043.1	AGTCTGCGAACTCATTTGGGAGGGCTAGTGGGGGGAGGGGGATGGAAATATTTAATAAATC
FQ312041.2	AGTCTGCGAACTCATTTGGGAGGGCTAGTGGGGGGAGGGGGATGGAAATATTTAATAAATC
FQ312044.2	AGTCTGCGAACTCATTTGGGAGGGCTAGTGGGGGGAGGGGGATGGAAATATTTAATAAATC
FQ312027.1	AGTCTGCGAACTCATTTGGGAGGGCTAGTGGGGGGAGGGGGATGGAAATATTTAATAAATC
FQ312045.1	AGTCTGCGAACTCATTTGGGAGGGCTAGTGGGGGGAGGGGGATGGAAATATTTAATAAATC
FQ312043.1	AGTCTGCGAACTCATTTGGGAGGGCTAGTGGGGGGAGGGGGATGGAAATATTTAATAAATC

Supplementary Table S7: Multiple sequence alignment of SP-PGQ-3

Consensus	TTCTTCGATCAGGTAGGAGAGCTCTTGGGCAACTTGGCTGGGGTCTAGTTCCACGGGACGGGAAGACTCAAGGAGAGTAAGGGTTTG
CP027540.1	TTCTTCGATCAGGTAGGAGAGCTCTTGGGCAACTTGGCTGGGGTCTAGTTCCACGGGACGGGAAGACTCAAGGAGAGTAAGGGTTTG
CP025256.1	TTCTTCGATCAGGTAGGAGAGCTCTTGGGCAACTTGGCTGGGGTCTAGTTCCACGGGACGGGAAGACTCAAGGAGAGTAAGGGTTTG
AP018391.1	TTCTTCGATCAGGTAGGAGAGCTCTTGGGCAACTTGGCTGGGGTCTAGTTCCACGGGACGGGAAGACTCAAGGAGAGTAAGGGTTTG
AP017971.1	TTCTTCGATCAGGTAGGAGAGCTCTTGGGCAACTTGGCTGGGGTCTAGTTCCACGGGACGGGAAGACTCAAGGAGAGTAAGGGTTTG
CP018347.1	TTCTTCGATCAGGTAGGAGAGCTCTTGGGCAACTTGGCTGGGGTCTAGTTCCACGGGACGGGAAGACTCAAGGAGAGTAAGGGTTTG
CP018138.1	TTCTTCGATCAGGTAGGAGAGCTCTTGGGCAACTTGGCTGGGGTCTAGTTCCACGGGACGGGAAGACTCAAGGAGAGTAAGGGTTTG
CP018137.1	TTCTTCGATCAGGTAGGAGAGCTCTTGGGCAACTTGGCTGGGGTCTAGTTCCACGGGACGGGAAGACTCAAGGAGAGTAAGGGTTTG
CP018136.1	TTCTTCGATCAGGTAGGAGAGCTCTTGGGCAACTTGGCTGGGGTCTAGTTCCACGGGACGGGAAGACTCAAGGAGAGTAAGGGTTTG
CP016227.1	TTCTTCGATCAGGTAGGAGAGCTCTTGGGCAACTTGGCTGGGGTCTAGTTCCACGGGACGGGAAGACTCAAGGAGAGTAAGGGTTTG
CP003357.2	TTCTTCGATCAGGTAGGAGAGCTCTTGGGCAACTTGGCTGGGGTCTAGTTCCACGGGACGGGAAGACTCAAGGAGAGTAAGGGTTTG
LN847353.1	TTCTTCGATCAGGTAGGAGAGCTCTTGGGCAACTTGGCTGGGGTCTAGTTCCACGGGACGGGAAGACTCAAGGAGAGTAAGGGTTTG
LN831051.1	TTCTTCGATCAGGTAGGAGAGCTCTTGGGCAACTTGGCTGGGGTCTAGTTCCACGGGACGGGAAGACTCAAGGAGAGTAAGGGTTTG
CP007593.1	TTCTTCGATCAGGTAGGAGAGCTCTTGGGCAACTTGGCTGGGGTCTAGTTCCACGGGACGGGAAGACTCAAGGAGAGTAAGGGTTTG
FQ312041.2	TTCTTCGATCAGGTAGGAGAGCTCTTGGGCAACTTGGCTGGGGTCTAGTTCCACGGGACGGGAAGACTCAAGGAGAGTAAGGGTTTG
FQ312044.2	TTCTTCGATCAGGTAGGAGAGCTCTTGGGCAACTTGGCTGGGGTCTAGTTCCACGGGACGGGAAGACTCAAGGAGAGTAAGGGTTTG
CP001845.1	TTCTTCGATCAGGTAGGAGAGCTCTTGGGCAACTTGGCTGGGGTCTAGTTCCACGGGACGGGAAGACTCAAGGAGAGTAAGGGTTTG
HE983624.1	TTCTTCGATCAGGTAGGAGAGCTCTTGGGCAACTTGGCTGGGGTCTAGTTCCACGGGACGGGAAGACTCAAGGAGAGTAAGGGTTTG
CP002176.1	TTCTTCGATCAGGTAGGAGAGCTCTTGGGCAACTTGGCTGGGGTCTAGTTCCACGGGACGGGAAGACTCAAGGAGAGTAAGGGTTTG
CP002121.1	TTCTTCGATCAGGTAGGAGAGCTCTTGGGCAACTTGGCTGGGGTCTAGTTCCACGGGACGGGAAGACTCAAGGAGAGTAAGGGTTTG
FQ312029.1	TTCTTCGATCAGGTAGGAGAGCTCTTGGGCAACTTGGCTGGGGTCTAGTTCCACGGGACGGGAAGACTCAAGGAGAGTAAGGGTTTG
FQ312027.1	TTCTTCGATCAGGTAGGAGAGCTCTTGGGCAACTTGGCTGGGGTCTAGTTCCACGGGACGGGAAGACTCAAGGAGAGTAAGGGTTTG
FQ312045.1	TTCTTCGATCAGGTAGGAGAGCTCTTGGGCAACTTGGCTGGGGTCTAGTTCCACGGGACGGGAAGACTCAAGGAGAGTAAGGGTTTG
FQ312043.1	TTCTTCGATCAGGTAGGAGAGCTCTTGGGCAACTTGGCTGGGGTCTAGTTCCACGGGACGGGAAGACTCAAGGAGAGTAAGGGTTTG
FQ312042.1	TTCTTCGATCAGGTAGGAGAGCTCTTGGGCAACTTGGCTGGGGTCTAGTTCCACGGGACGGGAAGACTCAAGGAGAGTAAGGGTTTG
FQ312039.1	TTCTTCGATCAGGTAGGAGAGCTCTTGGGCAACTTGGCTGGGGTCTAGTTCCACGGGACGGGAAGACTCAAGGAGAGTAAGGGTTTG
FQ312030.1	TTCTTCGATCAGGTAGGAGAGCTCTTGGGCAACTTGGCTGGGGTCTAGTTCCACGGGACGGGAAGACTCAAGGAGAGTAAGGGTTTG
CP001993.1	TTCTTCGATCAGGTAGGAGAGCTCTTGGGCAACTTGGCTGGGGTCTAGTTCCACGGGACGGGAAGACTCAAGGAGAGTAAGGGTTTG
CP000921.1	TTCTTCGATCAGGTAGGAGAGCTCTTGGGCAACTTGGCTGGGGTCTAGTTCCACGGGACGGGAAGACTCAAGGAGAGTAAGGGTTTG
CP000920.1	TTCTTCGATCAGGTAGGAGAGCTCTTGGGCAACTTGGCTGGGGTCTAGTTCCACGGGACGGGAAGACTCAAGGAGAGTAAGGGTTTG
CP000919.1	TTCTTCGATCAGGTAGGAGAGCTCTTGGGCAACTTGGCTGGGGTCTAGTTCCACGGGACGGGAAGACTCAAGGAGAGTAAGGGTTTG
CP000918.1	TTCTTCGATCAGGTAGGAGAGCTCTTGGGCAACTTGGCTGGGGTCTAGTTCCACGGGACGGGAAGACTCAAGGAGAGTAAGGGTTTG
AE007317.1	TTCTTCGATCAGGTAGGAGAGCTCTTGGGCAACTTGGCTGGGGTCTAGTTCCACGGGACGGGAAGACTCAAGGAGAGTAAGGGTTTG
AE005672.3	TTCTTCGATCAGGTAGGAGAGCTCTTGGGCAACTTGGCTGGGGTCTAGTTCCACGGGACGGGAAGACTCAAGGAGAGTAAGGGTTTG
CP001033.1	TTCTTCGATCAGGTAGGAGAGCTCTTGGGCAACTTGGCTGGGGTCTAGTTCCACGGGACGGGAAGACTCAAGGAGAGTAAGGGTTTG
CP000936.1	TTCTTCGATCAGGTAGGAGAGCTCTTGGGCAACTTGGCTGGGGTCTAGTTCCACGGGACGGGAAGACTCAAGGAGAGTAAGGGTTTG
CP000410.1	TTCTTCGATCAGGTAGGAGAGCTCTTGGGCAACTTGGCTGGGGTCTAGTTCCACGGGACGGGAAGACTCAAGGAGAGTAAGGGTTTG

Supplementary Table S8: Change in the T_m in the absence or presence of various buffers in comparison to that of K^+ buffer (50 mM) for the three essential PGQs.

#PGQ	Δ Tm (T_mK^+-$T_m$no_cation)	ΔT_m (T_mK^+-T_mNa^+)	ΔTm (T_mK^+-T_mLi^+)	Δ Tm (T_mK^+-T_mMg^{++})	Δ Tm (T_mK^+ (200mM)-T_mK+(50 mM)
SP-PGQ-1	3.89	2.55	0.73	-1.77	1.85
SP-PGQ-2	7.62	1.06	5.44	4.75	4.37
SP-PGQ-3	13.52	0.08	1.48	2.22	4.34

Supplementary Table S9: Template and primer sequences used for Taq polymerase stop assay.

PGQs	Template (5' – 3')	Reverse Primer (5' – 3')
SP- PGQ-1	GGGCAACTTGGCTGGGGTCTAGTTCCACGGGACGGGAAGACTAGCA	TGCTAGTCTTCCCG
SP- PGQ-2	TTTGGAGGGCTAGTGGGGGGAGGGGGAGATTACTT	AAGTAATCTCCCCC
SP- PGQ-3	TGGATAGGGCTAATAGGGAGAGCAGGGACGGGGCGAGATTCAC	GTGAATCTCGCCCC

Supplementary Table S10: Overlapping forward and reverse primers used in PCR based mutagenesis reactions for mTFP plasmid engineering.

PGQs	Forward Primer (5' – 3')	Reverse Primer (5' – 3')
SP- PGQ-1	GGGCAACTTGGCTGGGGTCTAGTTCCACGGGA CGGGATGGTGAGCAAGGGCGAGGA	CCCGTCCCGTGGAACTAGACCCCAGCCAAG TTGCCCAACAACCAGCACGTTGCCCA
SP- PGQ-2	GGGCTAGTGGGGGGAGGGGGTATATATATGG TGAGCAAGGGCGAGGA	ATATATACCCCCTCCCCCCACTAGCCCCAC AACAACCAGCACGTTGC
SP- PGQ-3	GGGCTAATAGGGAGAGCAGGGACGGGGATAT ATATGGTGAGCAAGGGCGAGGA	ATATATCCCCGTCCCTGCTCTCCCTATTAGC CCCACAACAACCAGCACGTTGC
